# Dosage Compensation in Females with X-Linked Metabolic Disorders

**DOI:** 10.3390/ijms22094514

**Published:** 2021-04-26

**Authors:** Patrycja Juchniewicz, Ewa Piotrowska, Anna Kloska, Magdalena Podlacha, Jagoda Mantej, Grzegorz Węgrzyn, Stefan Tukaj, Joanna Jakóbkiewicz-Banecka

**Affiliations:** 1 Department of Medical Biology and Genetics, Faculty of Biology, University of Gdańsk, Wita Stwosza 59, 80-308 Gdańsk, Poland; patrycja_juchniewicz@wp.pl (P.J.); anna.kloska@ug.edu.pl (A.K.); joanna.jakobkiewicz-banecka@ug.edu.pl (J.J.-B.); 2 Department of Molecular Biology, Faculty of Biology, University of Gdańsk, Wita Stwosza 59, 80-308 Gdańsk, Poland; magdalena.podlacha@ug.edu.pl (M.P.); jagoda.mantej@phdstud.ug.edu.pl (J.M.); grzegorz.wegrzyn@ug.edu.pl (G.W.); stefan.tukaj@ug.edu.pl (S.T.)

**Keywords:** X chromosome inactivation, XCI, metabolic disorders, inborn errors of metabolism, X-linked inheritance

## Abstract

Through the use of new genomic and metabolomic technologies, our comprehension of the molecular and biochemical etiologies of genetic disorders is rapidly expanding, and so are insights into their varying phenotypes. Dosage compensation (lyonization) is an epigenetic mechanism that balances the expression of genes on heteromorphic sex chromosomes. Many studies in the literature have suggested a profound influence of this phenomenon on the manifestation of X-linked disorders in females. In this review, we summarize the clinical and genetic findings in female heterozygotic carriers of a pathogenic variant in one of ten selected X-linked genes whose defects result in metabolic disorders.

## 1. Introduction

A current list of genes/loci mapped to the X chromosome consists of 862 entries [1]. Defects in fifty-five of these result in well-characterized metabolic disorders [2], usually producing dissimilar phenotypic effects in the two sexes. X chromosome inactivation is an epigenetic dosage compensation mechanism equalizing expression levels of X-linked genes in females (XX) and males (XY) might be a major factor accounting for this variation.

In autosomal single-gene diseases, the expression of the mutated allele with respect to the normal one can be characterized as dominant, co-dominant, or recessive, which determines the pattern of inheritance as autosomal dominant or autosomal recessive. Similarly, inheritance of “sex”-linked traits according to classical genetics follows an X-linked dominant or X-linked recessive pattern. In the former case, daughters of affected males are always affected and transmit the disorder to both sons and daughters, while in the latter, carrier women do not develop symptoms but pass the disorder to affected sons [3]. Most of the X-linked metabolic disorders are ascribed to the category of X-linked recessive disorders; however, many of them do not follow the mentioned rules of vertical transmission. Having reviewed literature on penetrance and expressivity in over 30 X-linked disorders, Dobyns et al. [3] came to the conclusion that due to highly variable penetrance in females, in the case of X-linked disorders, the use of terms dominant and recessive should be ceased and X-linked disorders be simply described as “following X-linked inheritance”.

Based on a literature search performed in PubMed for the key term “metabolic disorder X chromosome inactivation”, which resulted in 189 hits [4], we selected the 10 most frequently appearing X-linked genes whose pathogenic variants cause eleven metabolic disorders both in males and females. We did not include *MECP2*-related phenotypes defined as Rett syndrome, as the *MECP2* gene has long been considered lethal for boys and only in recent years few cases of males were reported and also because the metabolic aspects of its pathology remain vague [5]. Characteristics of the ten selected genes and the number of their mutations described to date are presented in Table 1 and Table 2, respectively. The aim of this review was to analyze the spectra of clinical phenotypes in female heterozygotes carrying mutations in these genes with regard to their X chromosome inactivation status and discuss the influence of this phenomenon on the female susceptibility to X-linked metabolic disorders.

## 2. Dosage Compensation

The sex determination mechanism in mammals is based on chromosomes, with the primary sex determinant for females being the presence of two X chromosomes and the presence of an X and a Y chromosome for males. The human X chromosome consists of about 156 Mb DNA (5% of the genome) that contains over 850 protein-coding genes with a diverse range of functions, while the Y chromosome is relatively gene-depleted, consisting of 57 Mb DNA that carries 65 coding genes mainly involved in sex determination and fertility [7,8]. Balanced expression of X-linked genes between both sexes is achieved by an epigenetically regulated process of dosage compensation known as X chromosome inactivation (XCI), which occurs in females early in development. In general terms, following XCI, transcription of X-linked genes is restricted to a single, active X chromosome (Xa) and inhibited on the other inactive X chromosome (Xi). The inactive X chromosome is characterized by (i) overall transcriptional inactivation (except for certain X-linked genes which escape inactivation, including *XIST*); (ii) heterochromatic condensation at the interphase of the cell cycle (visible as the Barr body); (iii) late replication during the S phase; (iv) DNA methylation of cytosine residues at CpG dinucleotides in the 5′ region of X-linked genes; (v) hypoacetylation of histone H4; (vi) expression of the *XIST* (X inactive specific transcript) gene located at the X-inactivation center [9].

**Figure 1:**
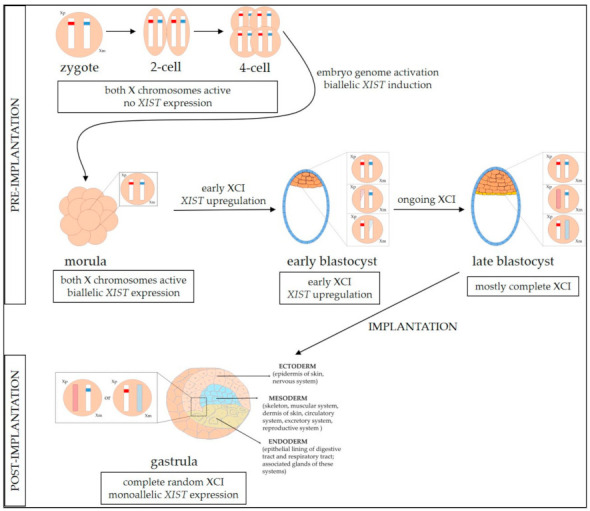
Model of X chromosome inactivation (XCI) in female human early development [14,15]. Female zygote inherits active maternal and paternal X chromosomes (Xm and Xp, respectively). Biallelic *XIST* expression starts from the four-cell stage concomitant with embryo genome activation and is upregulated in the blastocyst, in which progressive random XCI begins. The embryo at this stage consists of cells with either both X chromosomes active (white) or one X chromosome partially inactivated (red or blue). It is believed that complete XCI stabilizes between early implantation and the end of the first month of the pregnancy with different kinetics depending on cell lineage [16]. The XCI pattern established at the stage of gastrula and its three germ layers is stably maintained and clonally propagated through cell divisions. Nevertheless, the exact timing of complete XCI in humans remains elusive, as investigations past the blastocyst stage remain beyond current capabilities.

A study in a mouse model showed that XCI begins in cells of early female embryos at the X chromosome inactivation center (XIC), which comprises genetic elements crucial to this process: gene *XIST* and its cis-regulatory elements [10]. The long non-coding RNA *XIST* transcript coats the chromosome from which it was transcribed [11] and initiates interaction with multiple synergistic silencing pathways cooperating to maintain the silent state of the chromosome [12]. Recent findings on the mechanisms of dosage compensation in humans, based on single-cell RNA sequencing, led to the formulation of a model of an initially dual and partial expression dampening of the two X chromosomes [13]. However, Moreira de Mello et al., who re-analyzed the data presented in [13], argued against this model, showing evidence of an early XCI in human preimplantation development [14] (Figure 1).

Mary Lyon, who was the first to describe the phenomenon of dosage compensation in mice, proposed a random choice of which X chromosome will be silenced. According to this assumption, female somatic cells produce a mosaic consisting of two different cell lines, one with the Xi of paternal and the Xa of maternal origin and the other with a maternal Xi and paternal Xa in a 50:50 ratio [17]. A year later, the hypothesis of a random X-chromosome inactivation was extended to all mammals [18]. Deviations from this rule have been observed in marsupials, in which the X chromosome of paternal origin is always inactivated [19].

In some circumstances, deviations in the proportion of cell lines with inactivated X chromosomes of maternal and paternal origin are also observed in humans [20]. The phenomenon of skewing inactivation of the X chromosome does not have to be associated with the disease and can also occur in healthy women [21]. The most comprehensive population studies of 1005 healthy women and girls showed that the XCI pattern of ratio exceeding 70:30 was present in 25% of females, a ratio greater than 80:20 was found in 8%, while the ratio of 90:10 was identified only in 1.8% of the analyzed females [22].

Dosage compensation has two consequences that imply the differences between females and males in prevalence and severity of X-linked disorders. Firstly, irrespective of the number of X chromosomes, only one X chromosome is active in all cells of both sexes, and secondly, every female, regardless of being a genetic clone, is an epigenetic mosaic [23,24]. Opposite to most (over 90%) autosomal genes whose both parental copies are simultaneously expressed at comparable levels [25], X-linked genes are characterized by monoallelic expression. As mosaics, with some cells expressing proteins from the maternal X chromosome and the others from the paternal one, females have a more varied collection of cellular proteins than males whose cells express only the proteins encoded by the mother’s X chromosome. This cellular diversity gives females a biological advantage and enables them to ameliorate the effects of deleterious mutations of X-linked genes that lead to death or disease in hemizygous males. The female carrier of the pathogenic variant will not have it expressed in all her cells. Additionally, due to the cellular interactions known as metabolic cooperation, cells deficient in the functional protein may in many cases receive operational gene product secreted by normal neighboring cells.

Cellular interference is a rare phenomenon of abnormal cellular interactions occurring in craniofrontonasal syndrome (CFNS) that is an exceptional X-linked disorder with heterozygous females more severely affected than hemizygous males. CFNS is caused by the deficiency of ephrin-B1 (*EFNB1*), a transmembrane regulator of cell-cell communication playing an important role in boundary formation during development [26]. Human genetic studies have indicated that mosaicism for *EFNB1* mutation induced by random X inactivation in heterozygous females is central to CFNS pathology, and it was recently confirmed in human induced pluripotent stem cells (hiPSCs) model of the disorder [27]. It is assumed that another B-class ephrin may functionally replace deleterious ephrin-B1 in the homogenous cell population (in hemizygous males) but not when mutant and wild cell types coexist (in heterozygous females) [28]. However, the mechanisms underlying this action remain unknown.

The prevalence of genetic mosaicism during normal development and aging means that cell competition may occur as a result of somatic genetic differences in healthy people. Besides epigenetic mosaicism due to the XCI process, genetic large-scale chromosome X mosaicism (>2 Mb) was reported in blood and buccal samples of women at a frequency four times higher than that observed in the autosomes. It preferentially involved the inactive X chromosome, and its rate increased with age (0.11% in 50-year-olds; 0.45% in 75-year-olds) [29]. Contrary, in almost 200,000 men, genetic chromosome X mosaicism was detected at the lower level than for autosomes and was much rarer compared to females (0.006%), which reflects the importance of chromosome X gene dosage for cellular function and survival [30].

Depending on the XCI status, mutations on one of the X chromosomes might provide selective advantage or disadvantage. Elimination of one cell line is the most common way for two heterozygous cell populations to interact and is usually beneficial to women. Several genetic disorders may illustrate tissue-specific selection against cells with an active mutated X chromosome. For example, carriers of hypoxanthine phosphoribosyl transferase (*HPRT*) deficiency (Lesch–Nyhan syndrome) are almost completely devoid of mutant cells in their blood at the age of 10 years due to selection against HPRT-deficient erythrocyte precursors [23,31]. Heterogeneity of cell population in other tissues is nevertheless maintained since all but blood cells form gap junctions enabling metabolic cooperation between normal and HPRT deficient cells. As little as 5% of HPRT enzyme in females is sufficient to exert a protective effect against severe hyperuricemia observed in male patients [32]. As a consequence, heterozygous females rarely express any symptoms (including gout), while Lesch–Nyhan males have pathological uric acid deposits in many joints [23]. Other instances of cell selection in mosaic females may be found among X-linked immunodeficiencies. Wiskott Aldrich syndrome (WAS) is a primary immunodeficiency disease resulting in recurrent infections, eczema, and microthrombocytopenia in males and no clinical signs in female carriers. The lack of symptoms experienced by most heterozygous females may be related to a migration defect of hematopoietic progenitors and stem cells expressing mutant allele [33], which contributes to intensive cell selection early during hematopoietic differentiation.

Rarely, but sometimes, a mutation confers a proliferative advantage and results in cell selection that favors the mutant allele. If such cases applied to X-linked mutation, females should manifest symptoms of the disease that is usually only seen in males. The only known example of an X-linked disease in which the mutant allele has a selective advantage over the normal variant is adrenoleukodystrophy [32]. Proliferative advantage of mutant skin fibroblasts of carriers was shown in vitro; clones with an excess of long-chain fatty acids significantly outnumbered normal cells [23,34]. Predictably, the severity of disease in heterozygous females advances with age as women accumulate more of the mutant cells; in their 30s or 40s female patients develop adrenal myeloneuropathy, which is a milder form of the disease compared to males [23,34].

It is worth mentioning that in humans, up to 30% of X-linked genes escape transcriptional silencing, and since over half of them lack functional Y gametologs, their dosage remains imbalanced [35,36]. Expression of escape genes is sex-biased (usually female-biased) and links to prolonged lifespan, improved blood pressure regulation, and neuroprotection in females [35]. It also contributes to disease susceptibility, predisposing females to developing autoimmunity and males to certain cancers [35]. Posynick and Brown [36] pointed out that for the majority of over 140 X-linked intellectual disability genes, duplications, and consequent phenotypes have been detected, suggesting their substantial dosage sensitivity. Indeed, the dosage of some escape genes has been implicated in neurological phenotypes like seizures and Autism Spectrum Disorder [35].

X chromosome inactivation process and resultant degree of skewing is another important factor for the expression of genetic diseases. Skewing of X inactivation, where the X chromosome carrying a mutant allele is the most active X, has been observed in affected female carriers of several X-linked disorders. However, relationships between the pattern of XCI and clinical phenotypes are difficult to demonstrate [37]. Here we discuss how XCI affects phenotypic outcome in female heterozygous carriers of eleven X-linked metabolic disorders selected due to the amount of existing literature relating metabolic disorders to XCI.

## 3. X-Linked Metabolic Disorders

Inherited metabolic disorders (also known as inborn errors of metabolism) constitute an important group of genetic disorders characterized by disruption of cellular biochemical functions. Although individually rare, collectively, they represent a large and diverse class of conditions with a cumulative birth prevalence of 1 in 2000 live births [2,38]. In most of them, basic biochemical defect affects a metabolic pathway common to a considerable number of cells or organs giving rise to systemic consequences. This leads to very diverse presenting symptoms and secondary abnormalities, which can often render a diagnosis. Metabolic disorders can be classified in various ways. Saudubray and Charpentier [39] distinguished three groups of inborn errors of metabolism on the basis of their pathophysiology: (i) Disturbances in synthesis or catabolism of complex molecules (lysosomal and peroxisomal disorders; disturbances of intracellular trafficking and secretory protein processing), in which clinical symptoms are permanent, progressive, independent of intercurrent events, and unrelated to food intake; (ii) intoxication (aminoacidopathies, most organic acidurias, congenital urea cycle defects, and sugar intolerances), where a symptom-free interval is followed by clinical signs of acute or chronic intoxication and by recurrent metabolic disturbances; (iii) deficiency in energy production or utilization (glycogenoses, gluconeogenesis defects, congenital lactic acidemias, fatty acid oxidation defects, and mitochondrial respiratory chain disorders) with an overlapping clinical spectrum including hypoglycemia, hyperlactacidemia, severe generalized hypotonia, myopathy, cardiomyopathy, failure to thrive, cardiac failure, circulatory collapse, sudden infant death syndrome, and congenital malformations suggesting that the abnormal process affected fetal energy pathways.

Single gene metabolic disorders may follow different patterns of inheritance. Among 1015 recently identified well-characterized inborn errors of metabolism 922 (90.8%) were autosomal, 55 (5.4%) X-linked, 37 (3.6%) mitochondrial, and one digenic [2]. A lately proposed nosology of inborn errors of metabolism assigned metabolic disorders into 130 groups in nine (A–I) categories, and X-linked disorders could be found in all of these nine categories (Table S1) [2]. The disorders described in this review fall into seven distinct categories from this classification (Table 3).

**Figure 2:**
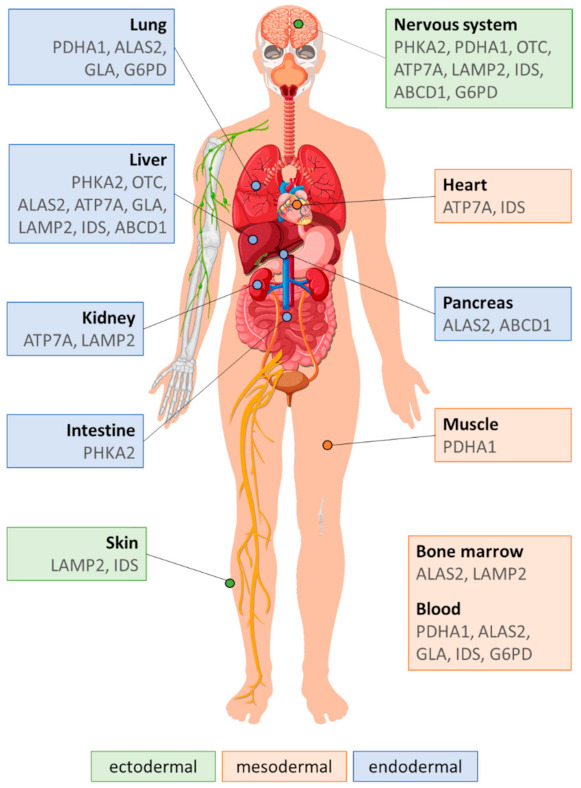
Tissues in which proteins are affected in the disorders described in this review are most abundantly produced and their embryonic origin. Tissue protein expression is based on data obtained from GeneCards^®^: The Human Gene Database; https://www.genecards.org/ (accessed on 27 January 2021) available as data integrated from literature manual curation, proteomics, and transcriptomics screens, and automatic text mining. Origin from germ layers is indicated for each tissue. Figure created using the human body figure designed by brgfx/Freepik (www.freepik.com; accessed on 27 January 2021).

Nine of ten genes related to the X-linked disorders described in this review code for enzymatic proteins, and one encodes a membrane-associated glycoprotein. Function, subcellular location, and tissue expression of these gene products are collectively shown in Table 4, while Figure 2 specifies the embryonic origin of tissues expressing genes selected for this work.

Defects in ten selected here genes result in eleven disorders that are briefly described below. Supplementary Materials (Tables S2 and S3) provide data about mutations, symptoms, or clinical severity, as well as XCI status in female patients described in the literature.

### 3.1. Glycogen Storage Disease Type IXa

Glycogen storage disease type IX (GSD IX) belongs to glycogen storage diseases, a group of inherited heterogeneous diseases characterized by abnormalities in glycogen metabolism and is caused by a deficiency in glycogen phosphorylase kinase (PhK) [40]. GSD IX accounts for 25% of GSD cases, and its most frequent subtype is GSD IXa [41]. The PhK enzyme activates liver and muscle glycogen phosphorylase, which is a key enzyme that mobilizes glucose from stored glycogen. PhK is composed of sixteen subunits (αβγδ)_4_. The liver isoform of the α-subunit performs a regulatory function controlled by phosphorylation and is encoded by the phosphorylase kinase liver alpha-subunit 2 (*PHKA2*) gene [42,43]. GSD IXa is caused by a mutation in the *PHKA2* gene [44].

GSD IXa has been biochemically divided into two further types: IXa1 with loss of PhK enzyme activity in peripheral blood cells and liver tissue, and IXa2 with normal PhK activity in blood cells. The clinical presentation of both subtypes is very similar [45,46]. Boys with GSD IXa typically present in the first two years of life with hepatomegaly, growth delay, elevated liver transaminases, increases in triglycerides and cholesterol, delayed motor development, chronic liver disease with cirrhosis and ketotic hypoglycemia [47,48]. These clinical and biochemical abnormalities gradually disappear with age, and most adult patients are asymptomatic [49].

The first reports of possible cases of X-linked GSD in females appeared in 1969 [50,51], and affected females were further suggested to be heterozygous [52]. In later years, very few cases of females affected by GSD IXa were reported [53,54], and XCI was shown to be the direct cause of the variable expression of the disease. Cho et al. described a case of GSD IXa manifested in a female Chinese patient accompanying a skewed XCI [53]. This patient showed phenotypic expression despite heterozygous status, which was due to preferential X inactivation with the wild-type allele. She had a history of mild liver enlargement, abdominal distension due to an enlarged liver, and deranged liver function. There were no significant abnormalities in blood glucose, lactate, pyruvate, urate, and triglycerides, but serum testosterone and dehydroepiandrosterone sulfate (DHEA-S) were elevated. During the course of the disease, the function and size of the liver gradually normalized. However, the patient complained of symptoms such as dysmenorrhea, menstrual irregularities and oligomenorrhea [53]. Another example of a female with mild GSD IXa symptoms has been reported in a scientific letter to the editor by Sanchez et al. [54]. Authors presented the clinical case of a girl whose relevant family history consisted of a brother with GSD type IXa and consanguinity. The female patient was diagnosed with hepatomegaly, reported no symptoms of hypoglycemia, and the adequate weight and height. Blood test showed elevated transaminase levels and levels of creatine phosphokinase, glucose, cholesterol, and triglycerides within normal ranges. An abdominal ultrasound scan showed diffuse changes in echogenicity. The authors suggested the need for carriage testing in women, as some females may develop GSD IXa symptoms of varying severity, which may be related to the degree of skewed XCI [54].

### 3.2. Pyruvate Dehydrogenase Deficiency

The pyruvate dehydrogenase complex (PDC) deficiency is a rare neurodegenerative disorder associated with abnormal mitochondrial metabolism. The PDC is a mitochondrial multienzyme complex that catalyzes the oxidative decarboxylation of pyruvate to acetyl-CoA, allowing connection between glycolysis and the tricarboxylic cycle and plays a vital role in carbohydrate metabolism and energy production [55]. PDC is composed of several copies of three catalytic elements: E1 (pyruvate dehydrogenase), E2 (dihydrolipoamide acetyltransferase), E3 (dihydrolipoamide dehydrogenase), and the non-catalytic E3-binding protein (E3BP). E1 is a thiamine diphosphate-depended enzyme consisting of two α and two β subunits (E1α and E1β). PDC activity is regulated by phosphorylation (inactivation) and dephosphorylation (activation) of three serine residues of E1α, performed by two enzymes: PDK (pyruvate dehydrogenase kinase) and PDP (phosphatase), which are also parts of the complex. The E1α is encoded by the *PDHA1* gene located on the X chromosome, and the remaining PDC components are encoded by autosomal genes [56,57]. Disease-causing mutations have been identified in E1β, E2, E3, and E3BP, and the regulatory enzyme PDP, but the majority of patients with PDC deficiency carry mutations affecting E1α [57,58].

PDC deficiency is commonly associated with lactic acidosis, progressive neuromuscular and neurological degeneration, and, often, death during childhood [58]. A wide spectrum of clinical presentations has been associated with PDC-E1α deficiency. In males, clinical symptoms can be categorized into four groups: (1) Neonatal lactic acidosis and encephalopathy, brain malformation, and facial dysmorphism; (2) childhood-onset progressive and chronic neurological deterioration; (3) with sometimes clinical and MRI findings similar to Leigh syndrome; (4) a childhood-onset milder or relapsing neurological disorder, with episodes of ataxia and weakness [57]. Females and males are almost equally affected, although the clinical characteristic differs between the sexes, and females have a milder disease course. The chronic neurological form predominates in females, while neonatal lactic acidosis is more common in males [59].

*PDHA1* is an X chromosomal gene, males are hemizygous, and all females identified so far are heterozygous for *PDHA1* mutations, which partially explains the significant difference in their clinical phenotype [59]. Heterozygous females with PDC deficiency carrying the same mutation could present a highly variable clinical phenotype. The pattern of XCI appears to be responsible for clinical variability in heterozygous females [56,59,60,61,62]. Dahl et al. [61] described three females with PDC-E1α deficiency from two unrelated families. All patients had the same mutation in the *PDHA1* gene; however, they differed widely in their clinical, biochemical, and pathological features. The authors suggested that variation in patterns of XCI in different females and between different tissues of the same individual may play an important role in generating this variability [61]. Another study showing that the pattern of XCI may influence the clinical phenotype in heterozygous females was carried out by Horga et al. [56]. They described two monozygotic twin females with the same heterozygous mutation in the *PDHA1* gene and clear differences in disease severity, which correlated well with residual PDC activity and XCI ratio [56]. Matthews et al. showed a correlation between the residual PDC activity, proportion of cells with immunoreactive E1α protein, and pattern of the XCI in heterozygous females with PDC-E1α deficiency [62]. Fujii et al. presented a heterozygous female patient with relatively mild symptoms of PDC-E1α deficiency and skewed inactivation of the mutant *PDHA1* allele. They observed a correlation between residual PDC activity, E1α immunoreactivity, the proportion of mutant cDNA, and the pattern of XCI in fibroblasts of the examined female [63]. Studies conducted in two unrelated heterozygous females with PDC-E1α deficiency and skewed XCI also supported the hypothesis that XCI might be the main factor in determining the clinical phenotype in females [64].

Diagnosis of PDC-E1α deficiency in females is difficult because deficiency of *PDHA1* can be biochemically undetectable due to the possibility of skewed XCI [57]. Matthews et al. [65] reported two heterozygous females with PDC-E1α deficiency with early-onset encephalopathy and lactic acidosis who had PDC activity within the normal range. Their XCI patterns in fibroblasts were 80:20 and 70:30 and were biased towards the expression of one allele (presumably the wild type) and correlated with the level of enzyme activity. The researchers emphasize that PDC activity was in the normal range in both females, despite severe central nervous system dysfunction and the only possible diagnostic method was molecular analysis, which identified a mutation in the *PDHA1* gene. The mainly affected tissue in patients with PDC deficiency is the brain, which is not accessible for biochemical analysis, so investigations are usually performed on cultured fibroblasts, which can lead to misdiagnosis in heterozygous females due to the process of XCI [65]. Willemsen et al. [66] described four unrelated heterozygous females with PDC-E1α deficiency. All of them showed skewed X inactivation (90:10) in their fibroblasts. Pyruvate dehydrogenase E1 subunit deficiency showed normal activity in three out of four patients; however, PDC-E1 deficiency was confirmed in muscle samples of all cases, indicating that enzyme measurements in fibroblasts are not always the appropriate method for the diagnosis of PDC deficiency in females due to the possibility of a skewed XCI [66].

### 3.3. Ornithine Transcarbamylase Deficiency

Ornithine transcarbamylase (*OTC*) deficiency is an X-linked inborn error of metabolism caused by mutations in the gene encoding ornithine carbamoyltransferase, which is a nuclear-encoded mitochondrial matrix enzyme that catalyzes the second step of the urea cycle in mammals. *OTC* deficiency is the most common urea cycle defect with estimated prevalence (biased toward the earliest and most severe presentations) from 1:14,000 to 1:77,000 live births [67].

Hemizygous males with complete *OTC* deficiency have a severe neonatal-onset disease and present with acute hyperammonemia within the first week of life. This is accompanied by cerebral edema, liver and renal failure, and early death if untreated [68]. A post-neonatal-onset disease occurs in males with partial deficiency of the enzyme and heterozygous females. Approximately half of these patients present later in life or even in adulthood [69], and their symptoms range from protein aversion only, through irritability and mild personality dysfunction, to profound neurological impairment and death secondary to encephalopathy [70]. Patients with milder *OTC* deficiency may not present until a hyperammonemia crisis is precipitated by stressors such as a severe illness, fasting, high protein meal, pregnancy, new medication, or even traffic accident [71]. While neonatal-onset disease in females is very rare [67], asymptomatic *OTC* deficient heterozygotes are the largest group of urea cycle disorders patients. It is estimated that approximately 80% of heterozygous females with *OTC* deficiency appear asymptomatic and present a normal biochemical workup [72]; however, even they have cognitive deficits and are at risk for learning disabilities and attention/executive function deficits [73]. Among a total of 908 *OTC* deficient patients enrolled in European and North American registries between February 2011 and January 2016, 556 (61%) were females (including asymptomatic carriers, whose number is unfortunately not presented in the reports) [74,75].

Therapeutic approaches to *OTC* deficiency aim to lower plasma ammonia levels, which can be achieved by the use of pharmacological ammonia scavengers combined with nutritional therapy (L-citrulline or L-arginine supplementation and a low-protein diet) [73]. An alternative to medical therapy is liver transplantation, performed typically by age six months in severe, neonatal-onset disorder. It is also considered in patients with milder phenotypes (both males and females) who have frequent episodes of recurrent hyperammonemia or poor metabolic status [72].

Currently, when clinically suspected, the diagnosis of *OTC* deficiency is most often confirmed by molecular genetic testing. More than 500 pathogenic *OTC* variants have been identified (Table 2). Almost two-thirds of them are nucleotide replacement at the coding region of the gene, and about 10% affect splicing of *OTC* mRNA. Table S2 presents the genotype-phenotype spectrum in the symptomatic *OTC* deficiency females; however, it omits cases without individual clinical descriptions like, for example, ones reported by Arranz et al. [76], Choi et al. [71], and Lu et al. [77]. Most symptomatic females carry missense mutations resulting in *OTC* destabilization, while there is a paucity of such mutations in males with late-onset *OTC* deficiency [32]. Gross deletions, frameshift, or nonsense mutations usually cause neonatal-onset disease in hemizygous males but not necessarily in females. Laróvere et al. [78] identified an Argentinian family with a complete deletion of the *OTC* gene, in which two symptomatic women with late-onset of disease and neurological involvement got pregnant. One of them developed decompensation during pregnancy and died, while the second remained stable and gave birth to a son with neonatal-onset disease who died in the neonatal period [78]. It indicates clearly that a type of mutation and biochemical properties of mutant *OTC* cannot be the only predicators of the *OTC* deficiency severity in heterozygous females. Among other determinants that need to be considered are environmental factors, genetic modifiers, and XCI patterns.

Although publications describing *OTC* deficiency in heterozygous females mention that variable clinical manifestations depend on the amount of XCI skewing, only a few have analyzed the lyonization patterns in carrier females. In 1976 Ricciuti et al. performed a histochemical in situ assay for *OTC* activity in a needle liver biopsy of an obligate *OTC* deficiency heterozygote and revealed two populations of hepatocytes; those with normal enzyme activity and those with no activity [79]. This first cytologic demonstration of cellular mosaicism for a liver-specific cell product confirmed X-linked inheritance of the disease.

Komaki et al. [80] were the first to perform PCR-based X inactivation analysis in a female with *OTC* deficiency. They described a case of a two-year-old girl who inherited a mutated *OTC* allele from her asymptomatic father with mosaicism and carrying both the wild-type and mutant alleles, as detected in peripheral leukocytes and spermatozoa. The XCI pattern was analyzed in the female patient’s liver biopsy specimen and showed preferential inactivation of the maternal X chromosome. At the age of 17 months, the girl presented with hepatomegaly. Then under dietary control, she developed normally until five years old when she suffered from acute respiratory infection, triggering a severe hyperammonemia attack leading to death [80].

Yorifuji et al. [81] analyzed the XCI patterns of peripheral blood leukocytes in a clinically normal mother and her two daughters with severe *OTC* deficiency manifestation, as well as five tissue samples were taken from various parts of the liver of one of these daughters who underwent the living-related liver transplantation from her father. Analysis of peripheral blood leukocytes revealed only a mild degree of skewing in the XCI pattern and no correlation between the degree of skewing and clinical phenotype—the phenotypically normal mother showed a slightly higher percentage of skewing than that of proband’s manifesting sister (64:36 vs. 54:46). Yet, samples of the proband’s liver had a higher degree of skewing than her leukocytes, and the degree of skewing differed from sample to sample (range 59:41 to 81:19), correlating well with the residual enzymatic activities of the samples [81].

A recent analysis of the XCI pattern in 25 samples of the explanted liver from a 14-year-old female patient with late-onset *OTC* deficiency showed remarkable intra-organ variation ranging from 46:54 to 82:18 in favor of the active X chromosome with the mutated allele of the *OTC* gene. These results were obtained by a standard DNA methylation-based assay and correlated with the *OTC* allele transcripts ratios assessed by deep parallel sequencing of RT-PCR products. The ratios in blood, saliva, and urine of the same patient ranged from 44:56 to 60:40 [68]. XCI analysis of 20 liver samples of another female *OTC* deficiency patient, who underwent liver transplantation at the age of 6 years, showed X inactivation ratio ranging from 75:25 to 90:10 in favor of the active paternal X chromosome presumably bearing de novo deleterious splicing mutation in *OTC* (c.663 + 1G > A). The ratio 75:25 was established in her blood [68]. Based on these outcomes, the authors suggested that liver biopsy analysis not be considered representative of the whole organ and that the X-inactivation status of peripheral blood leukocytes is completely irrelevant for predicting the status of the liver

### 3.4. X-Linked Sideroblastic Anaemia

X-linked sideroblastic anemia (XLSA) is the most common inherited form of sideroblastic anemia and is caused by deficiency of erythrocyte ALAS activity resulting in ineffective erythropoiesis and microcytic, hypochromic anemia [82]. XLSA results directly from loss-of-function mutations of the *ALAS2* gene [83]. More than 50 mutations that cause XLSA have been identified in the *ALAS2* gene, and most of them are missense mutations localized in exon 4 [84]. Decreased *ALAS2* activity leads to a reduction in protoporphyrin production and decreased heme. Iron, without control, enters the erythroblast and accumulates in the mitochondria [85]. The signs and symptoms of XLSA result from a combination of reduced hemoglobin and an overload of iron. Phenotypic expression of XLSA is highly variable even in patients with identical mutations. Symptoms of anemia or manifestations of parenchymal iron overload in affected males generally occur in the first decades of life [86]. The disorder is characterized by hypochromic microcytic anemia with ring-shaped sideroblasts in the bone marrow combined with systemic iron overload due to ineffective erythropoiesis [87]. During the course of the disease, patients develop hypersplenism, iron overload, and secondary hemosiderosis. Standard treatment for XLSA is based on high-dose pyridoxine supplementation and iron reduction strategies.

The majority of female carriers of XLSA are asymptomatic. However, occasionally heterozygous women with *ALAS2* mutations may be affected, and their phenotype may depend on the XCI pattern in hematopoietic tissue [88,89]. Aivado et al. described a family with XLSA in which a novel *ALAS2* mutation was identified in three women with a variable time of onset and clinical phenotype [88]. Their differing clinical course could be explained by the X-inactivation patterns of granulocytes and bone marrow cells. In affected women, the disease manifested as impaired hemoglobin production with erythrocytosis and hypochromia typically present in affected males. However, most females with XLSA have a macrocytic red blood cell phenotype that was also seen in the family described in that paper [88]. Interestingly, there were no cases of affected males in the analyzed family.

Cazzola et al. [89] studied a family with an elderly (age 71) woman who presented with acquired sideroblastic anemia. She was heterozygous for *ALAS2* mutation, and the mutated gene was expressed in her reticulocytes. Her two daughters and a granddaughter, who was heterozygous for this mutation, had normal hemoglobin levels. A grandson with a prior diagnosis of thalassemia intermedia was found to be hemizygous for the *ALAS2* mutation. All women who were analyzed in this family showed skewed X-chromosome inactivation in leukocytes with the preferentially active X chromosome carrying the mutant *ALAS2* allele [89]. Inactivation of the X chromosome in hematopoietic cells may play a role in the development of XLSA in female carriers with increasing age. Thus, the combination of congenital and acquired skewing may result in the late onset of XLSA in women. In conclusion, the results of this study make it clear that the most likely explanation of the above findings is that the proband, despite the apparently congenital imbalanced XCI in her hematopoietic cells, was able to produce normal amounts of red blood cells for the first six decades of her life, as her daughters and granddaughter. However, after the age of 70, she, like about 70% of older women, developed non-randomness of XCI. Unfortunately, the parental X chromosome with the normal *ALAS2* gene was inactivated, and when nearly all of the red blood cell precursors expressed the mutant gene, she developed severe anemia [89].

The next report presented a series of 29 probands suffering from XLSA [90]. Thirteen different *ALAS2* mutations were found in 16 probands (ten males and six females). Five of the six affected females had highly skewed X inactivation consistent with a diagnosis of XLSA (data not shown). One female was not informative for the X inactivation status [90].

Overall, in most of the reported cases of females with XLSA, affected male family members have not been encountered. Only a few reports have shown the same *ALAS2* mutation occurring in unrelated probands of both genders [87]. This suggests that in addition to X-inactivation skewing, the severity of the *ALAS2* mutation is an important determinant of disease expression in women and that the disease-causing mutation in females is often prenatally lethal in affected males.

Not all *ALAS2* mutations cause XLSA. Deletions or substitutions of the C-terminal 19–20 amino acids generate a truncated *ALAS2* protein with increased activity and result in X-linked erythropoietic protoporphyria cases [91,92,93].

### 3.5. X-Linked Protoporphyria

X-linked protoporphyria (XLEPP) is an extremely rare genetic disorder characterized by an abnormal sensitivity to the sun (usually beginning in infancy or childhood) that can cause severe pain, burning, and itching of sun-exposed skin. Its clinical manifestations are characterized not only by acute, painful, cutaneous photosensitivity but also by liver disease [94]. XLEPP belongs to the metabolic disorders of heme biosynthesis characterized by decreased iron stores and increased zinc- and metal-free protoporphyrin in erythrocytes. X-linked protoporphyria is an erythropoietic form of porphyria and is extremely similar clinically to erythropoietic protoporphyria (EPP) [95]. The diagnosis of XLEPP is suggested by generally higher concentrations of erythrocyte protoporphyrin, of which a high proportion is zinc-bound [93]. XLEPP results from gain-of-function mutations in the *ALAS2* gene that increase the activity of erythroid-specific delta-aminolevulinate synthase, the first enzyme in erythroid heme biosynthesis, in the bone marrow [96]. Due to the rarity of the disease, there are only a few reports in the literature on cases of XLEPP in women. The phenotype of heterozygous females can vary from asymptomatic to developing a severe form of the disease that is almost equal to that of affected males [84,86,87,88]. The median age of symptom onset for females with XLEPP was 11 years [97]. In heterozygous females, clinical variability is attributed to random X-chromosome inactivation.

The first literature report on the X-linked form of protoporphyria appeared in 2008 [93]. Whatley et al. [93] studied eight families in which at least one individual had clinically acute photosensitivity and deletion in the *ALAS2* gene. Both men and women had symptoms of the disease, presenting both photosensitivity and clinically overt liver disease. The authors found no evidence that X-inactivation led to milder disease in females. The concentrations of protoporphyrin in erythrocytes in photosensitive patients did not differ significantly between the sexes [93].

Brancaleoni et al. reported that 10 females who had the mutation in the *ALAS2* gene in the heterozygous state from six unrelated families of European descent and demonstrated that the XCI pattern directly influences the penetrance and the severity of the phenotype in females for XLEPP [98]. The authors showed markedly varying phenotypic and biochemical heterogeneity; two patients were fully affected with photosensitivity from childhood and increased erythrocyte protoporphyrin IX (PPIX), seven had later onset of symptoms, and only one had mildly increased PPIX, and one was completely asymptomatic. XCI studies on the females with mutations were consistent with the clinical and biochemical heterogeneity: both photosensitive females had a skewed pattern with over 73% of the wild type allele inactivated, the asymptomatic female had 88.4% inactivation of the mutant allele, and seven females with late-onset photosensitivity and mildly increased PPIX values had relatively balanced XCI patterns with preferential expression of the wild type *ALAS2* allele [98].

Twenty-two patients with XLEPP (10 men and 12 women) were examined in the next report [97]. Females had higher variability in clinical phenotype that ranged from symptoms appearing within 10 min of sun exposure to symptoms developing after prolonged exposure to the sun or even absence of symptoms. The clinical variability observed in patients with XLEPP was due to random X-chromosomal inactivation [97].

The latest update on the diagnosis and treatment of hereditary porphyria also included data on the prevalence of symptomatic forms of XLEPP in females. The authors of the report showed the occurrence of the first symptoms in the majority of females after 12 years of age and milder cutaneous manifestations compared to affected men [99].

### 3.6. Menkes Syndrome

Menkes disease (MNK) is caused by mutations in the *ATP7A* gene, the product of which is directly involved in copper metabolism [100]. One-third of the described clinical cases result from de novo mutations. Mutations in exon 17 result in the classic symptoms of this disease; however, the lack of a direct correlation between the type of mutation and the progression of the disease remains problematic [101].

MNK is a rare disease; its prevalence varies from 1 in 50,000 live births in Australia (resulting presumably from the founder effect) to 1 in 360,000 live births in Europe and Japan [102]. The vast majority of patients are males, but there are also some symptomatic females [101]. There are three main types of Menkes disease: classical MNK, mild MNK, and occipital horn syndrome (OHS). Classical MNK manifests in males in the first year of life with abnormal hair, convulsions, neurodegeneration, and lack of growth. In addition, without proper treatment, most patients die before the age of three. The OHS is dominated by connective tissue abnormalities, pathognomonic occipital exostoses, and mild mental retardation, usually occurring between the ages of 3 to 10 years. The mild (intermediate) type, in turn, is characterized by mental retardation, connective tissue abnormalities, *pili torti* (twisted hair shaft), cerebellar ataxia, and absence of seizures or childhood death. So far, only a few mild patients have been reported [100]. Irrespectively of the disease type, MNK patients may develop epileptic attacks, which can be divided into three periods. The early and intermediate stages are characterized by focal seizures and spasms. At a later period, in addition to those mentioned above, myoclonus is observed. Neuroimaging studies of MNK patients revealed brain atrophy, myelination, or demyelination, especially in temporal lobes. Detailed analysis showed characteristics similar to those observed in the course of stroke with mitochondrial myopathy and signs of oxidative stress. Neurological disturbances in patients with mild Menkes disease are limited to ataxia, dysarthria, moderate hypotonia, and mental development disorder. It should be noted that the occurrence of these symptoms is not the rule and varies between patients, which may be due to residual enzyme activity. Other signs of Menkes disease, which have diagnostic value, include hair abnormalities, including kinky and tangled hair, changes in bone and connective tissue, such as osteoporosis, exostosis, loose joints, and occipital horns [103]. A common problem, especially in younger patients, is pneumonia and recurrent respiratory infections [104]. Menkes disease is currently not curable, but with timely diagnosis and appropriate supportive therapy, its course can be milder and less burdensome for patients and their families [105].

Less than thirty MNK symptomatic females have been reported so far [106]. However, as some mildly affected females have not been characterized as manifesting carriers, the total number of females with any symptoms of MKN remains unknown [104]. Clinical features in the described female patients were generally milder, and life expectancy usually longer than that of affected males. The most frequently described symptoms in females included mental and motor retardation. The processes of learning and remembering new words were impaired, but the ability to understand them was usually at a normal level. In the severe course of the disease, accompanied by deviations in the electroencephalography (EEG) record such as rapid rhythmic waves and slow bilateral overlapping waves, retardation in mental development manifested as the inability to read, write, and speak even simple words. In such situations, a common form of communication was using pictograms [104]. Hair abnormalities such as *pili torti* and scattered hypopigmentation appeared in half of heterozygous females with MNK [106].

At least six Menkes-affected females had chromosomal aberrations—X-autosomal translocations or 45X/46XX mosaicism [104]. In two others, a mutation in *ATP7A* co-occurred with either two polymorphisms in this gene (c.2299G > C, IVS13-290A) or 500 kb deletion at Xq28 (not including the *ATP7A* locus) [106]. Interestingly, the third female patient described in the letter report carried no pathogenic mutations or deletions but only the two previously mentioned heterozygous polymorphisms in the *ATP7A* gene [106].

Møller et al. [104] studied potential associations between XCI, Menkes disease progression, and symptoms severity in nine affected heterozygous females with normal karyotypes. XCI patterns were also determined in unaffected carriers in their families (for details, see Supplementary Materials Table S2). The comparison of phenotypic disturbances with the XCI pattern showed a moderate correlation between the degree of X-chromosome inactivation and the level of mental retardation—a high degree of normal X-chromosome inactivation translated into a severe impairment of mental development, while preferential inactivation of the mutant X-chromosome associated with only moderate mental retardation [104]. Most unaffected carriers had skewed inactivation of the mutant X chromosome, as was also observed by Desai et al. [107]. Many authors emphasize that XCI patterns differ depending on the type of tissue analyzed. For example, the level of XCI measured in peripheral blood lymphocytes does not necessarily reflect the XCI status in the brain or skin [108]. To summarize, in some cases of females affected with MNK, symptoms correspond with the XCI patterns observed in peripheral blood lymphocytes; however, neither the type of mutation nor XCI pattern can predict the course of the disease and severity of symptoms.

### 3.7. Fabry Disease

Fabry disease (or Anderson–Fabry disease) is an X-linked lysosomal storage disorder caused by a deficiency of the enzyme alpha-galactosidase A (α-GAL A, GLA), which is required for the degradation of glycosphingolipids, mainly globotriaosylceramide (Gb3, GL3) [109,110]. The lack of enzyme activity results in the progressive accumulation of the substrate within lysosomes of cells in various organ systems and leads to irreversible damage to cells, tissues, organs, and systems dysfunction. Over a thousand mutations in the *GLA* gene have been described in Fabry patients. Most of these mutations are private, identified only in individual families. The incidence of Fabry hemizygotes is approximately 1:117,000 live births, but considering symptomatic heterozygotes, the incidence can be as high as 1:58,000 [111].

Fabry disease is characterized by multiorgan and progressive course. There are two major clinical phenotypes: the classical and the later-onset form. The classical form is mainly characterized in affected hemizygous males with absent or severely deficient *GLA* activity. The onset of the disease usually occurs during childhood or adolescence. Early manifestations include acroparesthesias, angiokeratoma, pain crises, hypohidrosis, gastrointestinal manifestations, and corneal/lenticular opacities. These symptoms worsen with age, and progressive GL3 accumulation leads to premature demise from renal, cardiac, and/or cerebrovascular disease. Males with later-onset phenotype have residual *GLA* activity and no vascular endothelial involvement. They develop renal, cardiac, or cerebrovascular manifestations between the fourth to eighth decades of life [110].

Heterozygous females show a wide spectrum of clinical signs and symptoms of Fabry disease. Usually, they have a later onset of symptoms, a slower rate of progression, and a higher phenotypic variability than male patients [112]. However, the life expectancy in female patients is also reduced, with cardiovascular disease being the most common cause of death both in females and males [113]. While the diagnosis of Fabry disease in male patients is usually established based on measurements of *GLA* activity in leukocytes, plasma, or cultured fibroblasts, the level of *GLA* activity in heterozygous females may be normal, decreased, or undetectable, leaving molecular analysis as the only reliable method for detecting female carriers [114]. However, the identification of female candidates to genetic testing may be facilitated by the use of a primary screening marker for Fabry patients of both genders, globotriaosylsphingosine (lyso-Gb3) [115].

The Mainz Severity Score Index (MSSI) is a common scoring system to assess the severity of Fabry disease. It consists of four components evaluating general, neurological, cardiovascular, and renal signs and symptoms of the disease in a patient. The obtained score allows classifying the severity of the disease as mild, moderate, or severe [116]. Table S3 shows the MSSI scores of female Fabry patients with different mutations and XCI status that have been described in the literature to date. Heterozygous females with Fabry disease showed mild to severe disease severity; furthermore, it progressed with age. The disease progression also varied between females carrying the same mutation, possibly due to the degree of XCI skewing. As the severity of the disease progresses with age, a modified scoring system, the Fabry Outcome Survey-MSSI (FOS-MSSI) [117], was proposed to allow the comparison of disease severity in different subgroups without sex or age confounding factors [118]. Another Fabry disease severity scoring system (DS3) was developed for easy use in the general Fabry patient population [119]; however, these scores are rarely reported in the literature for females with Fabry disease.

Disease manifestation in heterozygous females may be related to skewed XCI favoring the mutant *GLA* allele. Nevertheless, the results of studies assessing the occurrence of skewed XCI in females with Fabry disease are ambiguous. Some studies suggest a correlation between the severity of disease manifestation and XCI pattern [114,120,121,122,123], while others do not support these observations [112,124,125].

The study of XCI in monozygotic twins carrying the Fabry disease-causing mutation, one of whom was clinically affected and the other asymptomatic revealed opposite skewed XCI patterns (0:100 and 97:3) in fibroblasts, which seemed to explain the observed phenotypic difference between the twins [120]. Morrone et al. [114] described four Fabry disease female carriers from one Italian family. Two manifesting females showed a skewed XCI in favor of the mutant allele, while the other two asymptomatic females showed a skewed XCI in favor of the wild-type allele, suggesting a correlation between clinical manifestation and X inactivation in this family [114]. Out of 38 Czech and Slovak female patients, ten with skewed XCI favoring the mutant allele showed more rapid disease progression compared with one symptomatic female with skewed XCI favoring the wild-type allele and 27 symptomatic females with random XCI [121]. Bouwman et al. described a young female Fabry patient with the early and severe phenotype explained by the skewed XCI with 100% expression of the mutant allele [122]. Another study supporting XCI contribution to the morbidity of Fabry disease carriers was conducted in 56 female patients [123]. Skewed XCI ratio was detected in 16 (29%) of the studied females—10 of them predominantly expressed the mutant allele and six, the wild-type allele, suggesting that there are no selection mechanisms favoring the wild-type *GLA* allele. The authors observed that in heterozygotes with skewed XCI, disease progression was correlated with the direction of skewing. Females with preferential expression of the wild-type allele had a mild phenotype and little disease progression with age. In contrast, most females with preferential expression of the mutant allele were characterized by the early onset of disease, rapid progression with age, and poorer prognosis. Females with Fabry disease and random XCI (71%) showed disease manifestation worsening with age [123].

On the other hand, research results conducted on a group of 28 females with Fabry disease revealed no differences in the XCI ratio between these females and healthy females at the same age. Highly skewed X inactivation was observed in 18% of heterozygous females. These studies do not support the hypothesis that the disease manifestation in female carriers of the mutant *GLA* gene is related to skewed XCI [112]. Similar results were obtained by Elstein et al., who analyzed the XCI pattern in 77 heterozygous females, of whom only 18.2% had highly skewed XCI [125]. Another study showing that X inactivation is not a main contributor to the phenotype variability of Fabry disease in manifesting carrier females was carried out recently [124]. It was demonstrated that random XCI occurred in 91% of heterozygous females with a wide range of disease signs and symptoms while. one female with a slightly skewed XCI in favor of the mutant *GLA* allele had a relatively low disease severity [124].

### 3.8. Danon Disease

Danon disease is a multisystem disorder resulting from mutations in the lysosome-associated membrane protein-2 (*LAMP2*) gene. LAMP2 is a glycoprotein that coats the inner surface of the lysosomal membrane and is believed to protect it from proteolytic enzymes of lysosomes. A small cytoplasmic tail of the LAMP2 protein contains a lysosomal membrane targeting signal, enabling its activity as a receptor for proteins to be imported into lysosomes [126]. Alternative splicing of the terminal exon 9 produces three isoforms: LAMP2A, LAMP2B, and LAMP2C, varying in the sequence of the transmembrane and cytosolic tail and in their roles in autophagy [127]. Danon disease is classified as a form of autophagic vacuolar myopathy with intracytoplasmic autophagic vacuoles with sarcolemmal features [128].

Males with Danon disease typically present with the triad of hypertrophic cardiomyopathy, skeletal myopathy, and mild intellectual disability. Their survival beyond 25 years of age without heart transplantation is rare. In females, Danon disease should be considered if they present with either dilated or hypertrophic cardiomyopathy, retinal changes, mildly increased (or normal) creatine kinase (CK), aspartate aminotransferase (AST), and alanine aminotransferase (ALT) with preserved hepatic synthetic function [127]. Clinical features that occur in females approximately 15 years later than in males and are broader and more variable. However, cases of girls with unusual early presentation and severe disease course have also been described [129].

LAMP2 expression analysis was performed in different tissue samples from females related to boys with Danon disease. Sugie et al. [128] analyzed muscle biopsy from a woman with cardiomyopathy but without clinically apparent skeletal myopathy whose mentally retarded son developed progressive cardiomyopathy and mild skeletal muscle weakness. Immunohistochemical and Western blot analyses revealed a small amount of LAMP2 in her muscle despite a null mutation identified in the *LAMP2* gene (ten-base deletion at the junction of intron 5 and exon 6). Hashida et al. [130] described monozygotic twin sisters of a 13-year-old boy with left ventricular hypertrophy, muscle weakness in the upper extremities, and laboratory findings leading to Danon disease diagnosis. The girls did not exhibit any obvious symptoms of cardiac disease at the age of 11 years; however, in one of them, mild left ventricular hypertrophy was observed. They carried a 4 bp deletion in *LAMP2* at the intron 6 splice site (IVS6 + 1_4delGTGA). Immunohistochemical analysis showed chimerism of *LAMP2*-positive and *LAMP2*-negative monocytes, which was confirmed by fluorescence-activated cell sorting (FACS) of their leukocytes. In the latter analysis intensity of the signal differed in cell types. In both studies, reduced *LAMP2* expression in heterozygous female patients with Danon disease might result from random XCI; however, the patterns of XCI were not determined.

XCI pattern in Danon disease heterozygotic females was investigated only in few studies, most of which found random XCI pattern in a variety of tissues from different patients [126,129,131,132] (Table S2). Xu et al. [133] described one female *LAMP2* mutation carrier without clinical signs of Danon disease with skewed XCI, which might have helped her escape the negative influence of the mutation and protect her from developing symptoms.

### 3.9. Mucopolysaccharidosis II/Hunter Disease

Mucopolysaccharidosis type II (MPS II, also known as Hunter syndrome) is an X-linked lysosomal storage disease caused by the disturbed enzymatic activity of iduronate 2-sulfatase (EC 3.1.6.13), a lysosomal enzyme involved in the degradation of glycosaminoglycans (GAGs) [134]. The incidence of the disease is estimated at 1:68,000 to 1:320,000 live births and varies by population [135]. Mutations in the *IDS* gene encoding this enzyme resulting in a severe deficiency or complete absence of lysosomal iduronate 2-sulfatase activity leading to the impaired breakdown and excessive accumulation of dermatan sulfate and heparan sulfate [134]. Abnormal storage of GAGs in many cell types, tissues, and organs of affected patients is the primary cause of pathological changes and disease progression [134]. The diagnosis of MPS II is based on clinical features and biochemical outcomes. The disease symptoms include characteristic coarse facial features, short stature, characteristic bone, and joint abnormalities recognized on X-rays (dysostosis multiplex), enlargement of liver and spleen (hepatosplenomegaly), changes in the cardiovascular and respiratory systems; hearing and vision are commonly affected [136,137]. Neurological involvement, including developmental delay, progressive cognitive dysfunction, behavioral disturbances, and even seizures, is also recognized [136,137]. Diagnosis is always confirmed by low or absent iduronate 2-sulfatase activity detected in leukocytes, fibroblasts, or serum and elevated level of GAGs excreted in urine [137]. Noticeable differences of the disease phenotype expression are observed—the age of symptoms onset, clinical manifestation, and disease progression vary between affected individuals [137]. Despite the fact that a standardized system for severity scoring is absent, two extremes of the disease are distinguished based on clinical observations [136]. The severe type of the disease is recognized in the presence of progressive mental retardation, while mild or attenuated type—when intellectual development remains normal [137].

The *IDS* gene, due to alternative splicing events, gives several transcript variants and three different isoforms of iduronate 2-sufatase [138]. A large number of *IDS* sequence variants underlying the disease have been described so far (Table 2); substitutions resulting in missense or nonsense changes and small deletions are the most frequent types of mutations. As the *IDS* gene is localized on the X chromosome, mutations occurring within its sequence result in phenotype expression of MPS II primarily in hemizygous males; heterozygous females are usually unaffected carriers of the condition. However, symptomatic females are occasionally reported—to date; twenty cases were described in the literature. The first case of two females presenting symptoms of MPS II was reported in 1977 by Neufeld et al. [139], asking questions about possible mechanisms leading to the manifestation of disease in females. Later studies have shown that the XCI skewing is most often associated with the full expression of disease symptoms in females heterozygous for the *IDS* locus.

Heterozygous mutations in the *IDS* gene, accompanied by an extreme skewing of X-inactivation, leading to complete or nearly complete preferential inactivation of the X chromosome carrying the normal *IDS* allele, leaving the variant *IDS* allele active in all cells of a patient, have been detected in most of the female MPS II cases described in the literature (Table S2) [140,141,142,143,144,145,146,147,148,149]. Only one female with MPS II phenotype described to date resulted from homozygous mutation of the *IDS* gene, but the XCI skewing was also present in this case [142]. An interesting case of familial skewed XCI has been reported where brother and sister developed MPS II; the symptoms of the girl appeared due to preferential inactivation of the X chromosome bearing a fully functional allele [144]. Non-random XCI was detected in several female members of this family, including the mother of patients; however, the X chromosome with mutated *IDS* allele was preferentially inactivated in her cells. Pedigree of this family suggested that a potential mutation responsible for skewed XCI might be present in one of the autosomes [144]. Few other causes of female MPS II have been associated with chromosomal translocations [150,151], monosomy of the X chromosome [152], partial deletions of the X chromosome [153,154], or an intragenic inversion due to recombination between the *IDS* gene and its pseudogene [155]. These changes were also associated with extreme XCI skewing and preferential inactivation of the normal *IDS* allele.

Such heterozygous females with extreme XCI skewing and predominant expression of the variant *IDS* allele present the typical symptoms of MPS II (Table S2), not different from those observed in males with MPS II. Regardless of the type of mutation, female carriers with extremely skewed inactivation toward the normal *IDS* allele have very low or undetectable iduronate 2-sulfatase activity in serum, cultured fibroblasts, or other tissues tested, and excrete excessive amounts of GAGs in urine—biochemical measures typical of the MPS II phenotype. Mothers of MPS II female patients, who are also recognized as carriers, are reported as healthy. Only a few of them have been recognized with partial deficiency of iduronate 2-sulfatase enzymatic activity [140,141,153], but none have undergone a detailed medical examination.

To date, few studies assessed whether all female MPS II carriers, not only those with extreme XCI skewing, show any, even slight, signs and symptoms of the disease. Two clinical and biochemical studies showed that heterozygotes indeed have lower activity of iduronate 2-sulfatase in plasma and leukocytes comparing to non-carrier females [135,156]. Despite that, all female carriers in the study had normal levels of urinary GAGs excretion and were asymptomatic [135,156]. De Camargo Pinto et al. also did not find any differences in the X-inactivation pattern between these two groups [135]. Contrary, Guillén-Navarro et al. [157] observed some of the typical MPS II clinical manifestations in female carriers with moderately skewed, ranging between 75–86 percent, XCI—these females showed increased levels of urinary GAGs and presented skeletal anomalies, liver abnormalities, carpal tunnel syndrome or respiratory and ear problems [157]. The severity of symptoms was more evident when the degree of XCI skewing was higher and the female was older [157]. Unfortunately, none of these studies determined for every case whether the normal *IDS* allele was preferentially inactivated and variant allele (carrying a mutation) was preferentially active or the other way round.

As far, only the extreme X-inactivation skewing toward the normal *IDS* allele is recognized to result in full expression of the MPS II phenotype in female carriers. Small study populations, incomplete biochemical and clinical findings for females in the study, or lack of the control group with non-carrier females that are examined exactly the same way as the study group are the most evident limitations that make it difficult to determine the relationship between disease penetrance in female MPS II carriers with random XCI or moderate skewing.

### 3.10. Adrenoleukodystrophy

X-linked adrenoleukodystrophy (ALD) is the most common leukodystrophy but also one of the most complex metabolic disorders involving the central nervous system [158]. It is a monogenic disease caused by mutations in the *ABCD1* gene, which codes for peroxisomal transporter ATP-binding cassette subfamily D member 1 (ALDP). It is an important intermediary in the transport of very-long-chain fatty acids (VLCFA). Its malfunctions result in inhibition of VLCFAs degradation and consequently lead to the accumulation of various lipids in different tissues and organs [159]. Studies covering 489 ALD families showed de novo mutations in 4.1% of cases, and in less than 1%, gonadal or gonosomal mosaicism have been confirmed [160]. However, it was also estimated that the maternal risk for an additional ovum with a mutant allele found in an index case was at least 13%, indicating a significantly increased risk that the allele causing the disease will be transmitted to other offspring [160]. So far, over 680 mutations in the *ABCD1* gene have been described (Table 2). The vast majority of them are associated with an almost complete absence of ALDP, as they result in a reduction or lack of protein stability. There are two regions where most of these mutations occur—the first located between amino acids 83 and 344 (transmembrane domain region), and the second, between amino acids 500 and 668 (ATP-binding domain). All types of mutations that cause a complete lack of functional ALDP are found in patients showing a fully symptomatic X-ALD phenotype [161].

Historically, the first X-ALD cases were probably described in the late nineteenth century. In 1897 Heubner described a young boy with rapidly progressive neurological problems resembling X-ALD, which were classified as “diffuse sclerosis”. Schilder described several case reports with cerebral lesions accompanied by acute inflammatory changes. Since then, all symptoms analogous to those mentioned by Schilder have been called “Schilder’s disease” [162]. Even today, determining the actual number of cases still remains a problematic issue, mainly due to the lack of uniform diagnostic criteria. As we know, the disease was initially diagnosed only in boys before it was realized that similar symptoms described in adult men belong to the spectrum of X-ALD. Only a more detailed case study based on advanced diagnostic tools showed that women could also develop the full spectrum of symptoms. The X-ALD frequency, most frequently cited in the literature, is based on the results of a large US study. The estimated 1:42,000 cases is the sum of confirmed X-ALD cases in men in 1996–1998, divided by the number of all children born at the same time. The estimates described in the above study concerned only men. For frequency estimation of heterozygotes, the authors assumed that the ratio of heterozygotes to hemizygotes was 1.5. Thus, the combined estimated disease frequency for hemizygotes and heterozygotes was 1:16,800 [161]. In conclusion, the most common interpretation seems to be that X-ALD affects one person per 17,000 if both genders are taken into account or one in 21,000 live birth males. It is assumed that the number of cases is similar in all regions of the world and ethnic groups. For example, in France this is 1:100,000, while in Germany it is 0.8:100,000, and in the USA it is 1.6–3.6:100,000 [163].

Four ALD forms have been distinguished: pre-symptomatic, cerebral demyelinating inflammatory adrenoleukodystrophy (CALD), adrenomyeloneuropathy (AMN), myelopathy among women, and primary adrenal insufficiency only. Male patients most often suffer from adrenal insufficiency and myelopathy. Diagnosis is made on the basis of serum VLCFA levels and genetic testing [164]. The most common forms of X-ALD are CALD and AMN, found in populations around the world [162]. CALD only affects boys, usually between 5–14 years. It is associated with progressive inflammatory demyelination of the white matter of the brain, which in turn leads to deterioration of the functions of the neurological system, complete disability, and death at all ages [158]. The first symptoms to appear are destructive behaviors, aggressiveness, and learning problems, followed by aphasia, regression in writing and reading, impaired spatial orientation, visual disturbances, seizures [159]. Jiang et al. analyzed 19 cases of boys with CALD and found skin hyperpigmentation as the main symptom (53% of patients) [165]. On the other hand, AMN develops slowly and presents with axonopathy with initial clinical symptoms in men aged 20–30 years and women in the age range 40–50 years. In women, the symptoms are much milder [166]. The characteristic clinical picture of a man with AMN is impaired motor activity caused by stiffness and weakness in the legs. Additionally, adrenal dysfunction and impotence were observed. A diagnosis of AMN occurs within 3–5 years after presentation, unless a family history of X-ALD [165]. Treatment is only symptomatic; survival is estimated in decades. One of the first symptoms is VLCFA accumulate in the reticular and fasciculatory zones of the adrenal cortex, causing their insufficiency (reduced secretion of androgens and cortisol) [158]. Adrenal dysfunction may develop into AMN or remain the only symptom of X-ALD and may also develop into CALD [159]. Regarding female symptoms, Engelen et al. [166] conducted a study in which patients with X-ALD developed symptoms of myelopathy and/or peripheral neuropathy over the years. It was observed that the incidence of symptoms in women increased rapidly with age (from 18% of women > 40 years of age to 88% of women > 60 years of age), and men with X-ALD experienced high levels of VLCFA throughout their lives [166]. In women, genetic testing was necessary as 15% of X-ALD patients had normal VLCFA values [166]. In women patients, spinal cord disease is diagnosed later in life, and its progression is slower [167]. In addition to the clinical differences in spinal cord disease, there is a biochemical difference between the sexes. It has recently been discovered that 1-hexacosanoyl-2-lyso-sn-3-glycero-phosphorylcholine (C26:0-lysoPC) is a better diagnostic biomarker in women than C26:0. C26:0-lysoPC levels were elevated in 49 women, although C26:0 was not. Unfortunately, the difference between the C26:0-lysoPC through control level and the patient’s through C26:0-lysoPC level was small, justifying the need for a better discriminating biomarker [168]. Symptoms are highly likely to occur in patients with X-ALD. Age is the most important factor, and most patients have clinical signs over 60 years of age. It is mainly myelopathy. Peripheral sensorimotor neuropathy occurs with the frequency of 57% of respondents. X-linked adrenoleukodystrophy should be considered for diagnosis in women with chronic myelopathy [166]. There is no effective X-ALD therapy. Current therapies include dietary therapy with Lorenzo oil (a combination of erucic acid and oleic acid), hormone adrenal replacement therapy, and stem cell therapy in the case of a childhood form of disease. However, their effectiveness in the treatment of AMN and female heterozygotes, it is not clearly defined [167].

Studies on the correlations between X-chromosome inactivation and the symptoms of ALD in female carriers were conducted, among others by Watkiss et al. [169]. Peripheral blood leukocytes collected from twelve carriers, three of which showed neurological disturbances and nine were asymptomatic, were analyzed. Among the three symptomatic patients, only one had a highly skewed X-inactivation pattern; a similar level of XCI was also reported in two asymptomatic women. In turn, in vitro studies on fibroblasts cultures collected from three independent families revealed a selective advantage of the mutant allele [161]. Other experiments on blood leukocytes demonstrated skewed XCI in 68% of 22 ALD carriers, of which 32% described the level of inactivation as high and 36% as moderate. Inactivation occurred in all symptomatic carriers, and interestingly random inactivation was also reported in half of the asymptomatic females. On the basis of the conducted analysis, a significant correlation between the skewed XCI pattern and the severity of neurological symptoms was shown, but no associations between the level of inactivation and the VLCFAs concentration were observed. The problem in the described experiments is the fact that the DNA was obtained from the non-neuronal cells, such as skin fibroblasts or blood leukocytes, in which skewed XCI patterns may differ from those in nervous system cells [167].

### 3.11. Glucose-6-Phosphate Dehydrogenase Deficiency

Glucose-6-phosphate dehydrogenase (*G6PD*) deficiency is a common human enzymopathy caused by a mutation in the *G6PD* gene [170]. Its product consists of 515 amino acids that fold to form the monomer [171]. Depending on the NADP^+^ concentration, active human *G6PD* exists in a homodimer/tetramer (dimer of dimers) equilibrium [172]. The *G6PD* catalyzes the first step of the oxidative stage of the pentose phosphate pathway and produces NADPH. This nucleotide is indispensable to the recycling of glutathione (GSH) from its oxidized form (GSSG), which in effect leads to the elimination of reactive oxygen species through the reduction potential of reduced glutathione [173]. More severe *G6PD* pathogenic variants may result in the deformation of either the NADP^+^ binding or active site of the enzyme. Other changes in the sequence of this protein may hinder monomers interactions, disturb the protein structure, and decrease enzyme stability—all of which lead to an impairment in enzyme activity.

Approximately 400 million people suffer from *G6PD* deficiency, most in Africa and Asia [170]. To date, over 230 mutations in the *G6PD* gene have been described (mostly single-base missense mutations). Most of them result in reduced enzyme activity but not in a completely inactive enzyme which would be fatal to developing embryos [174,175,176].

World Health Organization (WHO) distinguished five classes of *G6PD* deficiency, depending on the level of enzyme activity: class 1 representing the most severe enzyme deficiency associated with chronic nonspherocytic hemolytic anemia; classes 2–4 comprising consecutively severe, moderate to mild, and very mild deficiency of enzyme activity; class 5 with the increased enzyme activity. Genetic variants belonging to different classes vary both in the intensity of clinical symptoms and the frequency of occurrence in different ethnic groups. Polymorphic variants in classes 3 and 4 (10–60% normal *G6PD* activity) are observed with increased frequency in some populations in Africa, Southeast Asia, the Middle East, and the Mediterranean region; however, the most common Mediterranean variant belongs to class 2 (<10% of normal *G6PD* activity) and beside the Mediterranean variant was also observed in the Middle East and India. It has been noticed that there may be more than one variant of gene mutation in the population [177,178]. Only a few de novo mutations have been found [179,180,181,182]. The diagnosis of the *G6PD* deficiency is based on the blood count and smear (CBC), qualitative and quantitative tests of the *G6PD* activity, or (PCR) [183,184,185,186].

There exists a fundamental difference in disease development between the sexes. Heterozygous females manifest a wider range of *G6PD* deficiency. Their cells deficient in *G6PD* are as prone to hemolysis as in male patients [187,188,189,190], so clinical symptoms in females vary depending on the amount of active enzyme and the percentage of expression of the abnormal allele in erythrocytes. By the degree of XCI skewing, three heterozygous female phenotypes arise 10% with normal, 80% with intermediate, and 10% with low enzyme activity [191,192]. Ultimately, as a result, most heterozygous females exhibit significantly fewer symptoms depending on the XCI pattern [188,190]. Homozygosity for the abnormal *G6PD* gene does not happen often, and these females are just as severely affected as men [193].

Corchia et al. [183] described a female newborn with symptoms of acute hyperbilirubinemia who turned out to suffer from *G6PD* deficiency. The hemolytic crisis occurred in the fetus after the mother consumed broad beans (regarded as the hallmark trigger for *G6PD* mutation carriers) a few days before delivery. At birth, the infant manifested clinical and hematologic signs of hemolytic anemia, hemoglobinuria, and no blood group immunization. Examination of *G6PD* activity revealed that both the infant and her asymptomatic mother were heterozygous for *G6PD* deficiency [183].

In a 31-year-old Filipino woman with dizziness, headache, dyspnea, generalized body edema, and hematuria, *G6PD* deficiency was diagnosed along with paroxysmal nocturnal hemoglobinuria (PNH) [193]. It was the third reported case of the coexistence of these two disorders, and the previous two were also women. PNH is a clonal disorder caused by mutations in the phosphatidylinositol glycan anchor (*PIGA*) gene involving hematopoietic stem cells. Both PNH and *G6PD* are very rare, and both are X-linked. What is more, erythrocyte hemolysis occurs in patients suffering from any of the two disorders; however, it is mediated through different mechanisms; thus, the authors of the report discus the facilitating effect of these conditions to each other and the possible effect of the state of dual morbidity on the response to treatment [193].

Au et al. [194] described two Chinese women who developed severe hemolysis related to *G6PD* deficiency at the age of 86 and 61 years after cotrimoxazole therapy [194]. The following investigation of the frequency of *G6PD* deficiency in women over 70 years old revealed 7/132 (5.3%) asymptomatic heterozygotes. All nine elderly *G6PD* mutation carriers had significantly more skewed XCI patterns (range 64:36–99:1, median 82:18) than ten younger ones identified in the study (range 52:48–67:33, median 56:44) who had normal enzyme levels [194]. Successive *G6PD* mutation analysis in 173 Chinese women older than 80 years identified 18 heterozygotes, of which three were biochemically *G6PD*-deficient [195]. XCI rates for the whole cohort ranged from 73:27 to 100:0, which confirmed previous observations that skewed XCI patterns are common in elderly women [196]. Au et al. suggested that XCI skewing with age might indicate the selective advantage of *G6PD*-normal red cells [195]. They also pointed out a relatively high frequency of mutant alleles in their female population—comparable with that of male newborns. Since in most female *G6PD* mutation carriers, hemolysis does not occur spontaneously but can be provoked by infection or drugs with oxidizing properties, e.g., most of the antimalarial agents, some sulfur drugs, and urinary antibiotics, it might be advisable to screen for *G6PD* activity before prescribing oxidative drugs to women over 60 [195].

It has been shown that *G6PD* deficient red cells inhibit the growth of *Plasmodium falciparum* malaria in vitro even when mixed with *G6PD* normal cells [197]. This proves that the increased incidence of *G6PD* deficiency in certain populations is due to the selective pressure of falciparum malaria and also that not only hemizygous males are protected from the parasite but heterozygotic females as well. What is more, the parasite growth rate in female *G6PD* mutation carriers should correlate with the percentage of normal cells in their blood, being thus dependent on their XCI pattern.

## 4. Summary of Effects of Skewed X Inactivation on Metabolic Diseases

The process of XCI is a critical event affecting the expression of X-linked genes in females. Despite the initial hypothesis, this process is not always random, resulting in an XCI pattern with 50:50 ratio of cell lines with inactivated X chromosome of maternal and paternal origin. Deviations in this proportion have been observed, and XCI status is defined as being highly skewed or extremely skewed as the preferential inactivation toward one of the X chromosomes exceeds 80% and 90% of cells, respectively [20,22,198]. Skewing of X inactivation, where the X chromosome carrying a mutant allele is the most active X, has been described in many symptomatic female carriers of X-linked disorders, including X-linked metabolic disorders described in this review. Female carriers of X-linked metabolic disorders manifest with varied phenotypes—from normal to affected with symptoms similar to those observed in disease-affected males. One might expect it depends on the amount of gene product to carry out the essential metabolic function produced by cells with active chromosomes carrying the normal allele [32]. However, a direct correlation between the severity of clinical symptoms in female carriers and the degree of X inactivation skewing is still difficult to assess, and many reports show opposite results.

Several limitations make it difficult to unequivocally determine the correlation of the X-linked disease penetrance in female carriers with the degree of X inactivation skewing. Analysis of XCI status performed in order to assess or predict the severity of an X-linked disease in a female carrier would give the most valuable result if the tested tissue was easily accessible and showed a high correlation of the XCI status with the disease-affected tissue. Peripheral blood is the most commonly used tissue for XCI ratio testing, but the correspondence of blood XCI status to that of disease-affected tissues remains undetermined for most of the X-linked metabolic diseases. In most studies, XCI status is determined in the most easily accessible clinical material, such as white blood cells, cheek epithelial cells, or saliva, rather than the appropriate tissue that is affected by the disease.

Although skewed XCI may explain why Fabry disease female carriers may manifest the disease symptoms, a consistent relationship between the degree of XCI skewing determined in tested tissue available from the patient (e.g., peripheral blood cells) and phenotype severity could not be clearly confirmed [112,125]. This was also the case in the family with ornithine transcarbamylase deficiency (*OTC*), where the XCI status in blood cells was random in female carriers, while it was skewed in the liver and correlated with the *OTC* activity [81].

The X inactivation skewing varies between tissues, ranging from 50% to 80% toward one of the X chromosomes in the same individual [199,200]. Moreover, cells with high mitotic activity characterized by greater XCI skewing patterns than cells with lower mitotic activity [190]. Yet, most of the studies on this process have determined the XCI status in samples taken from tissues composed of intensely dividing cells [20]. De Hoon et al. [201] investigated the correlation between several easily accessible tissues like blood, buccal swab, and hair follicles, and a number of inaccessible tissues such as thyroid, heart, liver, kidney, muscle, and ovary obtained from autopsy samples of 12 female individuals and concluded that buccal epithelium should be preferable over peripheral blood cells for predicting XCI pattern in inaccessible tissues. The ovary was the only inaccessible tissue showing a poor correlation to blood cells and buccal epithelium but had a good correlation to hair follicle instead [201]. The analysis of the XCI ratio for assessing or predicting the severity of an X-linked disease should take into account the degree of correlation between the tissue examined and the affected tissue, and this degree of correlation should be high.

The presence of secondary skewed XCI in the affected tissue only, without affecting other tissues, is an additional factor complicating phenotype predictions [201,202]. Secondary skewed XCI is the result of cell selection after inactivation that occurs at any age, in all tissues, or can be specific for a single tissue. It can result from a positive selection of cells with an inactivated X chromosome received from a particular parent. These cells acquire a survival advantage over cells that inactivated X chromosome obtained from another parent. Secondary skewed XCI can also result from a decreasing pool of stem cells during life and an increasing chance of most remaining stem cells having the same Xi.

In some X-linked metabolic diseases, intercellular communication known as metabolic cooperation is indicated as an important contributor to the phenotypic variability of disease manifestation in heterozygous females [23,32]. In general, cells bearing the normal allele of the gene on the active X chromosome produce and secrete a fully functional product, which may be provided to deficient cells unable to express this protein due to inactivation of the X chromosome carrying the normal allele. This phenomenon is also termed the cross-correction.

Most treatment modalities currently available for lysosomal storage disorders are based on the lysosome-specific cross-correction mechanism, as lysosomal enzymes are transferred between cells by mannose-6-phosphate-mediated endocytosis [203]. Nevertheless, the dissimilar extent of cross-correction efficiency may be at least partially responsible for different findings in MPS II and Fabry disease heterozygotes presented here.

The cross-correction in MPS II was shown to be efficient enough to reduce the storage in deficient cells by the uptake of the induronate-2-sulphatase enzyme secreted by normal cells [204]. That explains why the clinical manifestation of disease symptoms occurs only in heterozygous females with total XCI skewing favoring the mutant gene allele or in the absence of the normal allele due to chromosomal rearrangements while other female carriers with random or less skewed XCI are always asymptomatic. However, for Fabry disease, the cross-correction was experimentally shown to be less efficient. Fuller et al. [204] reported a lesser amount of α-galactosidase A secreted by unaffected cultured fibroblasts comparing to other lysosomal proteins. The cross-correction was not present between normal and Fabry disease skin fibroblasts in a co-culture. Moreover, only the mature form of α-galactosidase A, a form lacking the mannose 6-phosphate moiety, a tag necessary for efficient uptake via endocytosis by deficient cells, was detected in plasma. This limited cross-correction between cells due to inefficient enzyme uptake may thus greatly contribute to the manifestation of disease symptoms by Fabry heterozygous females even when the X-inactivation is random or only moderately skewed and cannot be associated with the phenotype variability.

Skewed XCI is common in the general female population, and about 35 percent of healthy females show XCI ratios of 70:20 or more skewed toward one of the Xs [22,205]. Skewing also tends to change with females’ age [144,206,207]. Longitudinal studies performed by Sandovici et al., in which two samples were collected at an average interval of two decades, showed a significant difference in the degree of XCI skewing with time in females who were 60 years or older at the time of the first sampling [208]. The biological significance of the age-related skewing of X inactivation is not clear. The skewed XCI in older females is hypothesized to be a consequence of advanced age though not related to their clinical situation. However, it may be responsible for the manifestation of X-linked disease symptoms at the more advanced age of a female carrier. Late-onset manifestations of X-linked glucose-6-phosphate dehydrogenase deficiency [194] and sideroblastic anemia [89] have been reported in elderly female carriers with skewed XCI. Once again, an evident question is the selection of tissues to be tested for XCI pattern in the case of female carriers of X-linked metabolic diseases in advanced age. As blood, usually used as a proxy for other tissues in determining XCI ratios, exhibits disproportionate to other tissues, age-related X inactivation skewing, it may not accurately reflect the effect of XCI skewing on the occurrence of symptoms from other organs specific to a given X-linked metabolic disorder [209].

The impact of XCI on the disease phenotype also varies between female carriers of different mutations in the disease-related gene. Therefore, the relationship between XCI status and the clinical phenotype may be misjudged when carriers with different mutations are pooled before analysis. In the diseases described in this review, there are cases of females from the same family and with the same mutation who had different clinical phenotypes.

Another limitation in assessing the contribution of XCI skewing on the phenotype in female carriers is the small number of investigated patients. The literature reports only single cases and limited studies performed on small groups of female carriers. Still, there is a need to assess the effect of skewed XCI in symptomatic females in studies on larger cohorts.

In addition to that, the method of XCI analysis and the skewed XCI definition itself varies between studies leading to misinterpretations. Different assays used to assess the XCI status can give various results. Most commonly, the analysis of XCI status is determined by assessment of the DNA methylation at the polymorphic androgen receptor (*AR*) locus (HUMARA assay). However, a direct comparison of this method with the method based on the analysis of allele-specific RNA expression at distinct heterozygous loci by RT-qPCR showed a number of inconsistencies suggesting that the methylation status does not always reflect expression [210]. Currently, other methods, e.g., integrated analysis of whole-exome and mRNA sequencing [211], allele-specific expression analysis [212], and genome-wide methylation analyses [213], are used in laboratory testing enabling a more accurate assessment of the XCI ratios.

## 5. Conclusions

Dosage compensation affects the phenotypic outcome in female carriers of X-linked metabolic disorders, but the relationships between the pattern of XCI and the severity of clinical phenotypes are still difficult to elucidate. Phenotypes of females are much more complex than just a result of the skewing in the XCI process and the percentage of unaffected cells in the relevant tissue. Selection against some cells, cellular interference, or the extent of cell-to-cell transfer of a gene product or metabolite greatly modify the clinical manifestation in heterozygous females, so that for some diseases, carriers usually stay asymptomatic while for others, they vary from asymptomatic to fully affected. Nevertheless, we believe that females with X-linked metabolic diseases in family history should always be examined in terms of their carrier status and pattern of XCI, including determination of which allele, the normal or disease-causing variant, is preferentially inactivated, to recognize or predict whether they will be symptomatic or not. Female carriers with moderate XCI skewing should be carefully monitored throughout their lives for the appearance or progression of any signs and symptoms of an X-linked metabolic disease that may occur with age.

## Figures and Tables

**Table 1:** Characteristics of genes whose mutations are associated with the disorders described in this review.

Disease (OMIM Number)	Gene Product Name	Gene Symbol	Number of			Chromosomal Location	
			Exons	Nucleotides ^1^	Amino Acids ^2^		
Glycogen storage disease type IXa (# 306000)	phosphorylase kinase regulatory subunit alpha 2	*PHKA2*	33	91,817	1235	Xp22.13	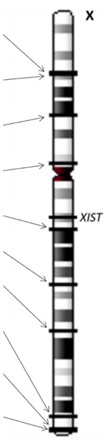
Pyruvate dehydrogenase deficiency (# 312170)	pyruvate dehydrogenase E1 alpha 1 subunit	*PDHA1*	12	17,813	390	Xp22.12	
Ornithine transcarbamylase deficiency (# 311250)	ornithine carbamoyltransferase	*OTC*	10	68,919	354	Xp11.4	
X-linked sideroblastic anaemia (# 300751)/Protoporphyria (# 300752)	5′-aminolevulinat synthase 2	*ALAS2*	12	21,923	587	Xp11.21	
Menkes syndrome (# 309400)	ATPase copper transporting alpha	*ATP7A*	23	139,703	1500	Xq21.1	
Fabry disease (# 301500)	galactosidase alpha	*GLA*	8	10,123	429	Xq22.1	
Danon disease (# 300257)	lysosomal associated membrane protein 2	*LAMP2*	9/10 *	43,202	410	Xq24	
Mucopolysaccharidosis II/Hunter disease (# 309900)	iduronate 2-sulfatase	*IDS*	11	28,319	550	Xq28	
Adrenoleukodystrophy (# 300100)	ATP binding cassette subfamily D member 1	*ABCD1*	11	19,905	745	Xq28	
Glucose-6-phosphate dehydrogenase deficiency/Hemolytic anemia (# 300908)	glucose-6-phosphate dehydrogenase	*G6PD*	14	16,180	515	Xq28	

^1^ Length of the gene in NCBI *Homo sapiens* Updated Annotation Release 109.20201120 on GRCh38.p13; ^2^ All amino acid positions coded in the gene including chain (extent of a polypeptide chain in the mature protein following processing or proteolytic cleavage), and for some genes also initiator methionine cleaved from the mature protein, N-terminal signal peptide, and a propeptide, which is a part of a protein that is cleaved during maturation or activation; * The human *LAMP2* gene has nine exons with the terminal one existing in two forms: 9a and 9b.

**Table 2:** Number of mutations in genes associated with the disorders presented in this review described in The Human Gene Mutation Database [6].

	*PHKA2*	*PDHA1*	*OTC*	*ALAS2*	*ATP7A*	*GLA*	*LAMP2*	*IDS*	*ABCD1*	*G6PD*
Missense/nonsense	65	108	342	86	125	692	35	331	425	213
Splicing	7	14	55	5	70	49	21	61	35	4
Regulatory	Nm	Nm	9	2	Nm	6	2	Nm	Nm	1
Small deletions	23	18	50	6	64	142	28	127	113	13
Small insertions	11	28	16	Nm	18	45	13	53	54	Nm
Small indels	Nm	2	5	1	1	16	1	15	16	2
Gross deletions	9	7	51	3	69	39	8	54	38	2
Gross insertions/duplications	Nm	19	2	1	24	8	2	4	1	Nm
Complex rearrangements	Nm	Nm	8	Nm	6	7	Nm	20	6	Nm
Repeat variations	Nm	Nm	nm	Nm	nm	Nm	Nm	Nm	Nm	Nm
HGMD Professional 2019.4 total/public * total	115/110	196/164	538/498	104/93	377/335	1004/844	110/74	665/552	688/652	235/211

* public entries are available for Academic/non-profit users only; Nm: no mutations.

**Table 3:** Nosology of the disorders described in this review according to Ferreira et al. [2].

Disease	Gene Symbol	Category and Group of Inborn Errors of Metabolism
Glycogen storage disease type IXa	*PHKA2*	C. Disorders of carbohydrates 51. Glycogen storage diseases
Pyruvate dehydrogenase deficiency	*PDHA1*	D. Mitochondrial disorders of energy metabolism 54. Disorders of pyruvate metabolism
Ornithine transcarbamylase deficiency	*OTC*	A. Disorders of nitrogen-containing compounds 7. Disorders of ammonia detoxification
X-linked sideroblastic anemia/Protoporphyria	*ALAS2*	F. Disorders of tetrapyrroles 98. Disorders of heme metabolism
Menkes syndrome	*ATP7A*	B. Disorders of vitamins, cofactors, metals, and minerals 40. Disorders of copper metabolism
Fabry disease	*GLA*	G. Storage disorders 102. Sphingolipidoses
Danon disease	*LAMP2*	C. Disorders of carbohydrates 51. Glycogen storage diseases
Mucopolysaccharidosis II/Hunter disease	*IDS*	G. Storage disorders 105. Mucopolysaccharidoses
Adrenoleukodystrophy	*ABCD1*	H. Disorders of peroxisomes and oxalate 110. Disorders of peroxisomal β-oxidation
Glucose-6-phosphate dehydrogenase deficiency	*G6PD*	C. Disorders of carbohydrates 49. Disorders of the pentose phosphate pathway and polyol metabolism

**Table 4:** Characteristics of proteins affected in the disorders described in this review.

Protein Symbol	Recommended Name ^a^	Function	Subcellular Location ^a^	Tissue Expression ^b^
PHKA2	Phosphorylase b kinase regulatory subunit alpha, liver isoform (EC: 2.7.11.19)	Phosphorylation of serine in certain substrates, including troponin I; possible calmodulin-binding	Plasma membrane	Nervous system, liver, and intestine
PDHA1	Pyruvate dehydrogenase E1 component subunit alpha, somatic form, mitochondrial (EC:1.2.4.1)	Conversion of pyruvate to acetyl-CoA and CO_2_ (link between glycolysis and the tricarboxylic acid cycle)	Mitochondrion matrix	Nervous system, muscle, liver, and blood
OTC	Ornithine carbamoyltransferase, mitochondrial (EC:2.1.3.3)	Synthesis of l-citrulline from l-ornithine and carbamoyl phosphate (urea cycle)	Mitochondrion matrix	Liver and nervous system
ALAS2	5-Aminolevulinate synthase, erythroid-specific, mitochondrial (EC:2.3.1.37)	Synthesis of 5-aminolevulinate from glycine and succinyl-CoA (heme biosynthetic pathway)	Mitochondrion matrix	Blood, liver, lung, and pancreas
ATP7A	Copper-transporting ATPase 1 (EC:7.2.2.8)	Cu(I) transport across cell membranes	Golgi apparatus, plasma membrane, cytosol (isoform 3), endoplasmic reticulum (isoform 5)	Liver, kidney, heart, and nervous system
GLA	Alpha-galactosidase A (EC:3.2.1.22)	Removal of the terminal α-galactose from glycoproteins and glycolipids (hydrolysis of glycosphingolipids)	Lysosome	Lung, blood, and liver
LAMP2	Lysosome-associated membrane glycoprotein 2	Chaperone-mediated autophagy; binding and targeting proteins for lysosomal degradation; fusion of autophagosomes with lysosomes; role in lysosomal protein degradation; required for MHCII-mediated presentation of exogenous antigens	Lysosome, endosome, and autophagosome membranes; plasma membrane	Nervous system, liver, bone marrow, kidney, and skin
IDS	Iduronate 2-sulfatase (EC:3.1.6.13)	Hydrolysis of the 2-sulfate groups of the L-iduronate 2-sulfate units of dermatan sulfate, heparan sulfate, and heparin (degradation of glycosaminoglycans)	Lysosome	Nervous system, skin, heart, liver, and blood
ABCD1	ATP-binding cassette sub-family D member 1 (EC:7.6.2.4)	Transport of free very-long-chain fatty acids (VLCFAs) and their CoA-esters across the peroxisomal membrane (regulation of VLCFAs and energy metabolism)	Endoplasmic reticulum, peroxisome, lysosome, and mitochondrion membranes	Pancreas, nervous system, and liver
G6PD	Glucose-6-phosphate 1-dehydrogenase (EC:1.1.1.49)	Oxidation of glucose-6-phosphate to 6-phosphogluconolactone, which reduces NADP^+^ to NADPH (pentose phosphate pathway)	Cytosol, membrane	Lung, blood, and nervous system

^a^ Data from UniProt Knowledgebase; https://www.uniprot.org/; accessed on 27 January 2021; full name recommended by the UniProt consortium; subcellular location of the mature protein according to UniProt manual annotation. ^b^ Data from GeneCards^®^: The Human Gene Database; https://www.genecards.org/; accessed on 27 January 2021; summary from section ‘Evidence on tissue expression from TISSUES’ representing tissue expression of a gene available as data integrated from literature manual curation, proteomics, and transcriptomics screens, and automatic text mining; sorted according to assigned confidence scores that facilitate comparison of the different types and sources of evidence; original source of information: TISSUES database; https://tissues.jensenlab.org; accessed on 27 January 2021. Terms with confidence scores between 5.0 and 4.0 are shown in this table.

## Data Availability

Not applicable.

## Supplementary Materials

The following are available online at https://www.mdpi.com/article/10.3390/ijms22094514/s1, Table S1: Fifty-five well-characterized X-linked metabolic disorders in nosology proposed by Ferreira et al., Table S2: Mutations and symptoms in symptomatic females suffering from ten disorders presented in this review, Table S3: Mutations and severity scores of heterozygous females with Fabry disease.
