# Supplementary material for: Dosage Compensation in Females with X-Linked Metabolic Disorders
Source: Int J Mol Sci. 2021 Apr 26;22(9):4514. doi: 10.3390/ijms22094514 (PMC8123450; doi:10.3390/ijms22094514)
Supplement: Supplementary file 1 [file ijms-22-04514-s001.zip › Supplementary File_Table S1.pdf]

Table S1. Fifty-five well characterized X-linked metabolic disorders in nosology proposed by Ferreira et al. [1]

Name	Group	Alternative names	Inheritance*	Gene	OMIM gene number
Category A. DISORDERS OF NITROGEN-CONTAINING COMPOUNDS					
Phosphoribosylpyrophosphate synthetase superactivity	2. Disorders of purine metabolism	-	XLR	*PRPS1*	311850
Phosphoribosylpyrophosphate synthetase deficiency	2. Disorders of purine metabolism	Arts syndrome (severe); X-linked Charcot-Marie-Tooth disease type 5 (intermediate); X-linked deafness type 1 (milder)	XLR	*PRPS1*	311850
Hypoxanthine guanine phosphoribosyltransferase deficiency	2. Disorders of purine metabolism	Lesch-Nyhan syndrome (severe); Kelley-Seegmiller syndrome (milder)	XLR	*HPRT1*	308000
Creatine transporter deficiency	4. Disorders of creatine metabolism	Cerebral creatine deficiency syndrome type 1	XL	*SLC6A8*	300036
**Ornithine transcarbamylase deficiency**	**7. Disorders of ammonia detoxification**	**-**	**XL**	***OTC***	**300461**
Monoamine oxidase A deficiency	10. Disorders of monoamine metabolism	Brunner syndrome	XLR	*MAOA*	309850
Ionotropic glutamate receptor AMPA type subunit 3 deficiency	20. Disorders of glutamate metabolism	Syndromic X-linked mental retardation, Wu type	XLR	*GRIA3*	305915
Category B. DISORDERS OF VITAMINS, COFACTORS, METALS AND MINERALS					

ABCB7 deficiency	25. Disorders of lipoic acid and iron-sulfur metabolism	Sideroblastic anemia and spinocerebellar ataxia	XLR	*ABCB7*	300135
Methylmalonic aciduria and homocystinuria, cblX type	26. Disorders of cobalamin metabolism	-	XLR	*HCFC1*	300019
**Copper-transporting ATPase α subunit deficiency**	**40. Disorders of copper metabolism**	**Menkes disease (severe); occipital horn syndrome (milder)**	**XLR**	***ATP7A***	**300011**
ATP7A-related distal motor neuropathy	40. Disorders of copper metabolism	X-linked distal spinal muscular atrophy type 3	XLR	*ATP7A*	300011
Category C. DISORDERS OF CARBOHYDRATES					
**Glucose-6-phosphate dehydrogenase deficiency**	**49. Disorders of the pentose phosphate pathway and polyol metabolism**	**-**	**XLR**	***G6PD***	**305900**
**Hepatic phosphorylase kinase α2 subunit deficiency**	**51. Glycogen storage diseases**	**Glycogen storage disease type 9a**	**XLR**	***PHKA2***	**300798**
**Lysosome-associated membrane protein 2 deficiency**	**51. Glycogen storage diseases**	**Danon disease**	**XL**	***LAMP2***	**309060**
Phosphoglycerate kinase deficiency	53. Disorders of glycolysis	-	XLR	*PGK1*	311800
Category D. MITOCHONDRIAL DISORDERS OF ENERGY METABOLISM					
**Pyruvate dehydrogenase E1-α deficiency**	**54. Disorders of pyruvate metabolism**	**-**	**XL**	***PDHA1***	**300502**
Pyruvate dehydrogenase kinase isoenzyme 3 superactivity	54. Disorders of pyruvate metabolism	-	XLD	*PDK3*	300906
NADH dehydrogenase α subcomplex subunit 1 deficiency	58. Disorders of complex I subunits	-	XLR	*NDUFA1*	300078
NADH dehydrogenase β subcomplex subunit 11 deficiency	58. Disorders of complex I subunits	Linear skin defects with multiple congenital anomalies type 3	XL	*NDUFB11*	300403

Cytochrome c oxidase subunit 7B deficiency	64. Disorders of complex IV subunits	Linear skin defects with multiple congenital anomalies type 2	XL	*COX7B*	300885
Holocytochrome c synthase deficiency	68. Disorders of mitochondrial cytochrome synthesis and incorporation	Linear skin defects with multiple congenital anomalies type 1	XLD	*HCCS*	300056
Taffazin deficiency	77. Disorders of mitochondrial phospholipid metabolism	Barth syndrome	XLR	*TAZ*	300394
TIMM8A deficiency	78. Disorders of mitochondrial protein import	Mohr-Tranebjaerg syndrome	XLR	*TIMM8A*	300356
USP9X deficiency	79. Disorders of mitochondrial protein quality control	X-linked mental retardation type 99	XL	*USP9X*	300072
AIFM1 deficiency	80. Other disorders of mitochondrial homeostasis	Combined oxidative phosphorylation deficiency type 6; Cowchock syndrome	XLR	*AIFM1*	300169
Category E. DISORDERS OF LIPIDS					
ε-N-trimethyllysine hydroxylase deficiency	82. Disorders of carnitine metabolism	-	XLR	*TMHLE*	300777
Glycerol kinase deficiency	87. Disorders of glycerol metabolism	-	XLR	*GK*	300474
Long-chain fatty acid-CoA ligase 4 deficiency	88. Disorders of cytoplasmic triglyceride metabolism	X-linked mental retardation 63	XL	*ACSL4*	300157
ZDHHC9 palmitoyltransferase deficiency	92. Disorders of palmitoylation	X-linked mental retardation, Raymond type	XLR	*ZDHHC9*	300646
Porcupine palmitoyltransferase deficiency	92. Disorders of palmitoylation	Goltz syndrome, focal dermal hypoplasia	XLD	*PORCN*	300651
Phosphatidylinositol 4,5-bisphosphate-5-phosphatase deficiency	93. Disorders of phosphoinositide metabolism	Lowe syndrome, Dent disease type 2	XLR	*OCRL*	300535

Myotubularin 1 deficiency	93. Disorders of phosphoinositide metabolism	X-linked myotubular myopathy	XLR	*MTM1*	300415
X-linked dominant sterol-4-alphacarboxylate 3-dehydrogenase deficiency	95. Disorders of cholesterol biosynthesis	Congenital hemidysplasia with ichythyosiform erythroderma and limb defects (CHILD) syndrome	XLD	*NSDHL*	300275
X-linked recessive sterol-4-alphacarboxylate 3-dehydrogenase deficiency	95. Disorders of cholesterol biosynthesis	CK syndrome	XLR	*NSDHL*	300275
X-linked dominant sterol Δ8-Δ7 isomerase deficiency	95. Disorders of cholesterol biosynthesis	X-linked dominant chondrodysplasia punctata type 2; Conradi-Hünermann-Happle syndrome	XLD	*EBP*	300205
X-linked recessive sterol Δ8-Δ7 isomerase deficiency	95. Disorders of cholesterol biosynthesis	Male EBP disorder with neurologic defects (MEND syndrome)	XLR	*EBP*	300205
Androgen receptor deficiency	96. Disorders of steroid metabolism	Androgen insensitivity syndrome	XLR	*AR*	313700
X-linked spinal and bulbar muscular atrophy	96. Disorders of steroid metabolism	Kennedy disease	XLR	*AR*	313701
Steroid sulfatase deficiency	96. Disorders of steroid metabolism	X-linked ichthyosis	XLR	*STS*	300747
Category F. DISORDERS OF TETRAPYRROLES					
**δ-aminolevulinic acid synthase deficiency**	**98. Disorders of heme metabolism**	**X-linked recessive sideroblastic anemia type 1**	**XLR**	***ALAS2***	**301300**
**δ-aminolevulinic acid synthase superactivity**	**98. Disorders of heme metabolism**	**X-linked protoporphyria**	**XLD**	***ALAS2***	**301301**
GATA1 deficiency	98. Disorders of heme metabolism	-	XLR	*GATA1*	305371
Category G. STORAGE DISORDERS					

WDR45 deficiency	100. Disorders of autophagy	Neurodegeneration with brain iron accumulation type 5; static encephalopathy of childhood with neurodegeneration in adulthood (SENDA); β-propeller protein-associated neurodegeneration (BPAN)	XLD	*WDR45*	300526
**α-Galactosidase A deficiency**	**102. Sphingolipidoses**	**Fabry disease**	**XL**	***GLA***	**300644**
**Iduronate sulfatase deficiency**	**105. Mucopolysaccharidoses**	**Mucopolysaccharidosis type 2; Hunter syndrome**	**XLR**	***IDS***	**300823**
Category H. DISORDERS OF PEROXISOMES AND OXALATE					
**X-linked adrenoleukodystrophy**	**110. Disorders of peroxisomal β-oxidation**	**-**	**XLR**	***ABCD1***	**300371**
Category I. CONGENITAL DISORDERS OF GLYCOSYLATION					
X-linked recessive UDP-Nacetylglucosamine transferase catalytic subunit deficiency	115. Disorders of N-linked glycosylation	ALG13-CDG	XLR	*ALG13*	300776
X-linked dominant UDP-Nacetylglucosamine transferase catalytic subunit deficiency	115. Disorders of N-linked glycosylation	Early infantile epileptic encephalopathy type 36	XLD	*ALG14*	300777
Oligosaccharyltransferase MAGT1 subunit deficiency	115. Disorders of N-linked glycosylation	MAGT1-CDG	XLR	*MAGT1*	300715
Translocon-associated protein δ subunit deficiency	115. Disorders of N-linked glycosylation	SSR4-CDG	XLR	*SSR4*	300090
O-linked N-acetylglucosamine transferase deficiency	119. Disorders of O-GlcNAcylation	X-linked mental retardation type 106	XLR	*OGT*	300255

PIGA-CDG	122. Disorders of glycosylphosphatidylinositol biosynthesis	Multiple congenital anomalies-hypotonia-seizures syndrome type 2; GPI biosynthesis defect type 4; early infantile epileptic encephalopathy type 20	XLR	*PIGA*	311770
UDP-galactose transporter deficiency	122. Disorders of glycosylphosphatidylinositol biosynthesis	SLC35A2-CDG; early infantile epileptic encephalopathy 22	XLD	*SLC35A2*	314375
ATP6AP1-CDG	129. Disorders of Golgi homeostasis	Immunodeficiency type 47	XLR	*ATP6AP 1*	300197
ATP6AP2-CDG	129. Disorders of Golgi homeostasis	X-linked mental retardation, Hedera type	XLR	*ATP6AP 2*	300556

* XLR, X-linked recessive; XL, X-linked; XLD, X-linked dominant; the disorders described in the revie are presented **in bold.**

References

1. Ferreira, C.R.; van Karnebeek, C.D.M.; Vockley, J.; Blau, N. A proposed nosology of inborn errors of metabolism. *Genet. Med.* **2019**, *21*, 102–106, doi:10.1038/s41436-018-0022-8.
