# Supplementary material for: Dosage Compensation in Females with X-Linked Metabolic Disorders
Source: Int J Mol Sci. 2021 Apr 26;22(9):4514. doi: 10.3390/ijms22094514 (PMC8123450; doi:10.3390/ijms22094514)
Supplement: Supplementary file 1 [file ijms-22-04514-s001.zip › Supplementary File_Table S2.pdf]

Table S2. Mutations and symptoms in symptomatic females suffering from ten disorders presented in this review (Fabry disease is not included in this tables; Fabry disease is presented in Table S3).

Mutation			Symptoms	XCI status	Ref.
DNA level	Protein level	Additional information			
**Glycogen storage disease type IXa (*PHKA2*)**					
c.1404dupT	p.His469SerfsTer12	duplication resulting in frame shift and premature termination	hepatomegaly, elevated transaminase levels	not determined	[1]
c.3614C>T	p.Pro1205Leu	substitution/missense	mild hepatomegaly, deranged liver function, no significant abnormalities in blood glucose, lactate, pyruvate, urate and triglycerides, elevated serum testosterone and DHEA-S, menstrual irregularity and oligomenorrhea	skewed	[2]
**Pyruvate dehydrogenase deficiency (*PDHA1*)**					
c.379C>T	p.Arg127Cys	substitution/missense	psychomotor retardation, hypotonia, spasticity, microcephaly, cerebral atrophy	90:10	[3]
c.484C>T[a]	p.Arg127Trp	substitution/missense	mental retardation, truncal ataxia, dysarthria	skewed	[4]
c.926dupTACC	no data	frameshift in exon 9	psychomotor retardation, hypotonia, seizures, cerebellar atrophy	90:10	[3]
c.949A>C[a]	p.Met282Leu	substitution/missense	dysmorphic features, hypotonia, brain atrophy, ventriculomegaly, frontoparietal cystic lesion	80:20	[5]
c.968G>A	p.Arg288His	CpG dinucleotide region of the E1α important for the interaction of α and β subunits	static encephalopathy with spastic quadriplegia, microcephaly, seizures, hypocalcaemia	skewed	[6]
c.900A>G[a]	p.Arg288ins	frameshift and premature termination	developmental delay, hypotonia, cerebral atrophy	70:30	[5]
no data	p.Ser300del20	frameshift	severe CNS and systemic biochemical dysfunction, neurodevelopmental abnormalities with a systemic lactic acidosis, progressive brain degeneration with neurologic deterioration	85:15	[7]

c.1009C>T	p.Arg302Cys	CpG dinucleotide region	severe neurological dysfunction, degenerative changes, developmental anomalies in the brain, variable lactic acidosis	skewed	[7]
c.1009C>T	p.Arg302Cys	CpG dinucleotide region	mild to moderate mental retardation and seizures in an adult	skewed	[8]
c.1100A>T	p.His367Leu	alteration of the conformation of the C-terminus of E1α and its interaction with E1β within the E1α2β2 heterotetramer	developmental delay, episodes of hypotonia or encephalopathy, epilepsy, slowly progressive motor impairment	55:45	[9]
c.1100A>T	p.His367Leu	alteration of the conformation of the C-terminus of E1α and its interaction with E1β within the E1α2β2 heterotetramer	similar (as above), but milder symptoms	75:25	[9]
c. 1132C>T	p.Arg378Cys	substitution/missense	psychomotor retardation, hypotonia, spasticity, seizures, microcephaly	90:10	[3]
c.1133G>A	p.Arg378H	substitution/missense	psychomotor retardation, hypotonia, ataxia, spasticity, seizures	90:10	[3]
c.1263ins12	no data	frameshift	static encephalopathy with spastic quadriplegia, microcephaly, seizures	skewed	[6]
**Ornithine transcarbamylase deficiency (*OTC*)**					
dupE1-9/delE10	-	chromosomal rearrangement: exons 1-9 duplication; exon 10 deletion; *de novo* mutation	late presentation with neurological involvement; frequent decompensations due to infectious processes; the patient developed a severe crisis that led to cerebral edema, cardiorespiratory arrest, and death at 2 years	not determined	[10]
c.103insA	p.Val35SerfsTer7	frameshift	recurrent vomiting accompanied with lethargy, death at 2 years	not determined	[11]
no data	p.Ile53Phe	substitution/missense	failure to thrive, IgA deficiency, malaise, headache, vomiting, altered mental status; liver transplantation at 9 years	not determined	[12]
c.268A>G	p.Ser90Gly	substitution/missense	mild symptoms such as nausea or vomiting; hepatic OTC activity <20%	not determined	[13]

c.270T>G	p.Ser90Arg	substitution/missense	recurrent vomiting, irritability	not determined	[11]
c.317G>A	p.Gly106Glu	substitution/missense	severe symptoms such as lethargy or convulsion more than four times a year; hepatic OTC activity <20%; liver transplantation at age 16 (after this, her clinical manifestations completely resolved and her serum ammonium level normalized)	not determined	[13]
c.365A>T	p.Glu122Val	substitution/missense	Initial symptoms: fever, cough, anorexia, and lethargy; presenting symptoms: mechanical ventilation, shock condition, deep coma, both dilated pupils at 4mm with weak light reaction; cerebral edema; sister of a boy who died at age of 4 days due to coma and dyspnea	not determined	[14]
c.368delG	p.Ser123IlefsTer	frameshift	recurrent vomiting, irritability, light coma	not determined	[11]
no data	p.Leu139Ser	substitution/missense	protein intolerance	not determined	[15]
c.421C>T	p.Arg141X	nonsense – premature termination	recurrent vomiting accompanied with lethargy; death at 7 years 10 months	not determined	[11]
c.422G>A	p.Arg141Gln	substitution/missense	feeding intolerance, irritability, altered sensorium; moderate developmental delay/learning disability, recurrent decompensation	not determined	[16]
no data	p.Arg141Gln	substitution/missense	poor growth, intermittent vomiting, oral aversion, gross motor delay, and altered mental status	not determined	[12]
c.444G>T	p.Leu148Phe	substitution/missense	enzyme activity 17%	no data	[17]
c.444G>C	p.Leu148Phe	substitution/missense	hepatomegaly; normal physical and mental development under dietary control until 5 years when she died of severe attack of hyperammonemia associated with acute respiratory infection; daughter of an asymptomatic father carrying the wild-type and mutant alleles	skewed	[18]
c.452T>G	p.Leu151Arg	substitution/missense	late presentation with neurological involvement and stable clinical outcome	not determined	[10]
c.482A>G	p.Asn161Ser	substitution/missense	recurrent vomiting accompanied with lethargy, irritability, partial seizure, avoidance of meat; death at 4 years 5 months	not determined	[11]

c.483T>A	p.Asn161Lys	substitution/missense	moderate symptoms such as lethargy or convulsion less than three times a year; hepatic OTC activity <10%	not determined	[13]
no data	p.Glu171Arg	substitution/missense	ataxia, lethargy, refusal to eat meat and diary products; recurrent hyperammonemic episodes; liver transplantation at 8 years and 3 months	not determined	[12]
c.533C>T	p.Thr178Met	substitution/missense	late presentation with neurological involvement; persistent vegetative state	not determined	[10]
c.540+1G>A	-	splice site error	late presentation with neurological involvement; stable outcome	not determined	[10]
c.540+2T>C	-	splice site error	recurrent vomiting accompanied with lethargy	not determined	[11]
c.583G>C	p.Gly195Arg	substitution/missense; additionally the patient is heterozygous for a polymorphism c.137A>G (p.K46R) located on the same chromosome as the mutation	first attack of hyperammonemia at 5 years and repeated attacks with altered states of consciousness during respiratory or gastrointestinal infections; liver transplantation at the age of 14 years	liver (25 samples): proportion of cells with active X chromosome with the mutation ranged 46:54-82:18; blood, saliva, and urine: the range was 44:60-60:40	[19]
no data	p.Asp196Val	substitution/missense	protein intolerance, migraine	not determined	[15]
c.595A>C	p.Asn199His	substitution/missense	recurrent vomiting, difficulty in breathing following a febrile illness; mild learning disability, infrequent decompensation on a low-protein diet and oral ammonia scavengers; her younger brother died of hyperammonemic coma at the age of 40 days	not determined	[16]
c.1001C>A[a]	p.Leu201Met	substitution/missense	complex partial seizures; death at 5.3 years	not determined	[11]
no data	p.Ser203Pro	substitution/missense	acute onset of vomiting, lethargy, irritability, and ataxia; liver transplantation at age 10 years; sister of a brother who died at 2 months of age	not determined	[12]
c.622G>A	p.Ala208Thr	substitution/missense (alanine to threonine substitution within a highly conserved domain)	feeling ‘generally unwell’ for many years; intermittent symptoms of nausea and lethargy; a fatally affected brother and son, who died aged 41 and 7 years respectively	not determined	[20]

c.626C>T	p.Ala209Val	substitution/missense	recurrent vomiting, irritability, psychomotor retardation, talipes valgus	not determined	[11]
c.628A>C	p.Lys210Gln	substitution/missense (*de novo* mutation)	severe neonatal-onset (at 60 h of life she began to be irritable and hypertonic with tachypnea and tachycardia; blood chemistry indicated respiratory alkalosis and mild polycytaemia; her conditions worsened to deep coma with clinically dominant circulatory failure, tachycardia and hypotension)	not determined	[21]
c.638T>C	p.Met213Thr	substitution/missense	vomiting, seizures and progressive acute encephalopathy following a febrile illness; died during the initial hyperammonemic episode at the age of 14 years	not determined	[16]
c.638T>C	p.Met213Thr	substitution/missense	history of protein avoidance; postpartum delirium; mildly symptomatic, nausea if consuming high protein content foods; mother of a daughter and son with OTC deficiency who died during their first hyperammonemic encephalopathy at 14 years and 2 days, respectively	not determined	[16]
no data	p.Met213Thr	substitution/missense	global developmental delay and poor oral intake; presenting symptom: intermittent vomiting at the age of 17 months	not determined	[12]
c.643C>T	p.Leu215Phe	substitution/missense	liver disfunction; enzyme activity 17%	not determined	[17] [22]
no data	p.Ala217fsTer229	frameshift	recurrent vomiting and protein aversion, recurrent hyperammonemic episodes; liver transplantation at 5 years and 5 months of age, epilepsy	not determined	[12]
c.663+aG>A	-	splice site error (*de novo* mutation)	Several metabolic decompensations and disturbances of consciousness; diagnosis at 5 years; persistent hyperammonemic episodes despite therapy initiation; liver transplantation at 6 years	liver (20 samples): proportion of cells with active paternal X chromosome (presumably bearing the	[19]

				mutation) ranged 75:25-90:10; blood: 75:25	
no data	p.Pro225Leu	substitution/missense	speech delay and poor oral intake, altered mental status; recurrent episodes of hyperammonemia	not determined	[12]
c.717G>A	p.Glu239Glu	donor splice site error	intermittent vomiting and failure to thrive	not determined	[12]
c.717+1G>A	-	donor splice site error	Initial symptoms: vomiting, anorexia, and lethargy; presenting symptoms: coma, no paralysis, no convulsion; expanded family history	not determined	[14]
c.718_2A>G	-	splice site error	recurrent vomiting accompanied with lethargy, irritability	not determined	[11]
c.813-814delAGinsC	p.Glu271Aspfs*288	frameshift	history of failure to thrive, hypotonia, seizures; acute encephalopathy and respiratory distress following a febrile illness; died during the initial hyperammonemic episode at 1 year	not determined	[16]
c.893G>A	p.Trp298Ter	nonsense – premature termination	recurrent vomiting	not determined	[11]
1033T>G[a]	p.Tyr313Asp	substitution/missense	episodic vomiting, seizures, myoclonic jerks, died at age of 33 months after developing coma; in 10 different sections of her liver biopsy OTC activity ranged 3-16% of normal	not determined	[23]
no data	p.Arg320Leu	substitution/missense	episode of coma; mother of a neonatal-onset affected sonfchoi	not determined	[15]
no data	p.Ala336Val	substitution/missense	episode of hyperammonemic decompensation associated with abnormal liver function	not determined	[12]
c.1005+1G>A	-	donor splice site error E9/I9	recurrent vomiting, irritability; death at 4 years 3 months	not determined	[11]
no data	-	G->A substitution at splice site of I9/E10	lethargy, coma; OTC activity in liver 23% of normal; 16 years of education; sister to a neonatal-onset affected brother	not determined	[15]
deletion of whole *OTC* gene			recurrent vomiting accompanied with lethargy, avoidance of meat and milk, psychomotor retardation, hypotonia, death at 1 year 9 months	not determined	[11]

	deletion of whole *OTC* gene		late presentation with neurological involvement; decompensation during pregnancy and death	not determined	[10]
	deletion of whole *OTC* gene		late presentation with neurological involvement; stable outcome; mother of a son with neonatal onset (who died in neonatal period)	not determined	[10]
**X-linked sideroblastic anemia (*ALAS2*)**					
c.509G4A	p.Arg170His	substitution/missense	sideroblastic anemia; presence of sideroblasts in the bone marrow	skewed	[24]
c.653G>A	p.Arg218His	substitution/missense	sideroblastic anemia; presence of sideroblasts in the bone marrow	skewed	[24]
c.679C>T	p.Arg227Cys	substitution/missense	macrocytic anemia, mild splenomegaly	not determined	[25]
c.1108G>A	p.Ala370Thr	substitution/missense	weakness, lower extremity edema, shortness of breath, amenorrhea, diabetes, ring sideroblasts	not determined	[26]
c.1184G>A	p.Cys395Tyr	substitution/missense	breathlessness and fatigue, diagnosed with severe microcytic anemia at the age of 64	skewed	[27]
c.1232G4A	p.Arg411His	substitution/missense	sideroblastic anemia; presence of sideroblasts in the bone marrow	skewed	[24,28]
c.1355G>A	p.Arg452His	substitution/missense	microcytic anemia with ring sideroblasts; diabetes mellitus II; myocardial infarction; hypercholesterolaemia	not determined	[29]
c.1358C>T	p.Arg436Trp	substitution/missense	macrocytic anemia, presence of numerous ring sideroblasts	marrow erythroid cells were nearly extremely skewed, extreme skewing in granulocytes and buccal mucosa	[30]
c.1559C4T	p.Pro520Leu	substitution/missense	sideroblastic anemia; presence of sideroblasts in the bone marrow	skewed	[24,31]
**X-linked protoporphyria (*ALAS2*)**					
c.1642C>T	p.Gln548Ter	premature termination	variable degree of phototoxicity; symptoms beginning after 13 years of age	not determined	[32]

c.1706-1709 delAGTG	p.Glu569Glyfs*24	deletion resulting in frame shift and premature termination	photosensitivity with pain, burning, and itching of sun-exposed skin; higher erythrocyte protoporphyrin levels and circulating fluorocytes	skewed	[32,33]
**Menkes syndrome (*ATP7A*)**					
c.-22-?del	-	deletion in exon 1	mild mental retardation at the age of 14 years; mild hypopigmentation, moderately coarse hair; her brother suffered from classical Menkes syndrome and died at 25 months	not determined due to homozygous *AR* locus	[34]
c.-22-?del	-	deletion in exon 1	unaffected carrier	100:0 (mutated:normal)	[34]
c.532G>T	p.Glu178Ter	nonsense – premature termination	convulsions at the age of 2 years, skeletal changes and ataxia at the age of 13; psychomotor delay, IQ of 83 at the age of 29 years	100:0 (mutated:normal)	[34]
c.532G>T	p.Glu178Ter	nonsense – premature termination	unaffected carrier	100:0 (mutated:normal)	[34]
c.532G>T	p.Glu178Ter	nonsense – premature termination	unaffected carrier	74:26 (mutated:normal)	[34]
c.1554-?_1707+?del	-	deletion in exon 6	frequent diarrhea and motor development delay in first months of life; was never able to walk independently; no language (uses pictograms to communicate) at the age of 12 year	not determined due to homozygous *AR* locus	[34]
c.1554-?_1707+?del	-	deletion in exon 6	unaffected carrier	90:10 (mutated:normal)	[34]
c.1554-?_2172 +?del	-	deletion in exon 6_9	mild mental and motor retardation	80:20; 78:22 (mutated:normal)	[34]
c.1554-?_2172 +?del	-	deletion in exon 6_9	unaffected carrier	98:2 ((mutated:normal)	[34]
c.1554-?_2172 +?del	-	deletion in exon 6_9	unaffected carrier	100:0 (mutated:normal)	[34]
c.1554-?_2172 +?del	-	deletion in exon 6_9	unaffected carrier	100:0 (mutated:normal)	[34]
c.1554-?_2172 +?del	-	deletion in exon 6_9	unaffected carrier	100:0 (mutated:normal)	[34]

c.1554-?_2172 +?del	-	deletion in exon 6_9	unaffected carrier	73:27 (mutated:normal)	[34]
c.1554-?_2172 +?del	-	deletion in exon 6_9	unaffected carrier	96:4 (mutated:normal)	[34]
c.1946+5G>A	IVS8+5G>A	splicing intron 8	could only express single words; motor retardation; convulsion; ataxia; failure to thrive; hypotonia; skeletal changes; hypopigmentation; abnormal hair; her brother suffered from classical Menkes syndrome and died at 6 months	24:76 (mutated:normal)	[34]
c.1946+5G>A	IVS8+5G>A	splicing intron 8	unaffected carrier	100:0 (mutated:normal)	[34]
c.1946+5G>A	IVS8+5G>A	splicing intron 8	unaffected carrier	98:2 (mutated:normal)	[34]
c.2179G>A	p.Gly727Arg	substitution/missense exon 10	severe mental retardation at the age of 2 years; motor retardation; unable to walk at 41 years; hypotonia, abnormal hair, hypopigmentation; her brother suffered from classical Menkes syndrome and died at 27 months	4:96 (mutated:normal)	[34]
c.2383C>A	p.Arg795Ter	nonsense – premature termination - exon 10	severe mental and motor retardation; convulsions; abnormal hair	4:96 (mutated:normal)	[34]
c.2383C>A	p.Arg795Ter	nonsense – premature termination - exon 10	unaffected carrier	92:8; 100:0 (mutated:normal)	[34]
c.2383C>A	p.Arg795Ter	nonsense – premature termination - exon 10	unaffected carrier	100:0 (mutated:normal)	[34]
c.4123+5G>	IVS21+5G>A	splicing intron 21	significant learning difficulties (IQ64 at 10 years); abnormal hair; hypopigmentation	51:49 (mutated:normal)	[34]
c.4123+5G>	IVS21+5G>A	splicing intron 21	unaffected carrier	78:22 (mutated:normal)	[34]
unknown	unknown	unknown	deafness at the age of 2 years; significant mental retardation – unable to read and write; no language; unable to walk without aid; 34 years old; her unaffected mother also showed highly skewed XCI pattern but with opposite X chromosome inactive	0:100 (based on real time RT-PCR, where ATPA7A transcript was absent leading to the assumption	[34]

				that the normal allele was inactivated)	
**Fabry disease (*GLA*) – see Table S3**					
**Danon disease (*LAMP2*)**					
c.277G>A	p.Gly93Arg	substitution/missense; mutation accompanied by *DSC2* p.G790del mutation	absence of clinical signs; nearly normal *LAMP2* expression	leukocytes: 82:18	[35]
c.294G>A	p.Trp98Ter	nonsense – premature termination - exon 10	palpitations and easy fatigue since 26 years; cardiac transplantation at the age of 52 years; mother of an affected son who was diagnosed with hypertrophic cardiomyopathy at 28 years of age	skeletal muscles (3 samples): 62:38, 58:42, 78:22; leukocytes (3 samples): 62:48, 48:62, 69:31	[36]
c.453delT	p.151fs	frameshift; mutation accompanied by intronic alteration in *LMNA*	severe hypertrophic cardiomyopathy; mild intellectual impairment since childhood; cardiac transplantation at the age of 23 years	leukocytes: 62:38; left ventricle: 56:44; septum: 61:39	[37]
c.795C>A	p.Cys265Ter	nonsense – premature termination	palpitations and breathlessness on exertion developed at the age of 39 years; after one year she became worse and had ICD implanted; she continued to deteriorate and underwent heart transplantation at the age of 41 years	not determined due to insufficient amount of the frozen material	[38]
c.808dupG	p.Ala270GlyfsTer3	frameshift	completely normal mother of a son with Danon disease in whom easy fatigability and decreased motor capacity began at 12 years of age	leukocytes (3 samples): 67:33, 69:31, 72:28	[39]
c.865-3C>G	p.Lys289fsTer36	aberrant splicing, frameshift and premature termination	cardiac hypertrophy; no muscle weakness or ataxia; heart transplantation at the age of 13 years	not determined due to insufficient amount of the frozen material	[38]
c.940delC	p.Ala314fsTer32	mutation putatively introduces 31 novel amino acids prior the appearance of a premature STOP codon.	diagnosed with hypertrophic cardiomyopathy without left ventricle outflow obstruction at age 15 years; at 26 mitral valve replacement; heart transplantation at 29; no abnormalities in skeletal muscle biopsy	preferential inactivation of the wild-type allele in leukocytes	[40]

c.973delC	p.Leu325fsTer21	frameshift and premature termination	cardiac hypertrophy; normal motor function; lower skeletal muscle strength; heart transplantation at the age of 10 years	not determined due to homozygous *AR* locus	[38]
**Mucopolysaccharidosis II / Hunter disease (*IDS*)**					
c.246T>C	p.Leu41Pro	substitution/missense They postulate that the patient is homozygous for p.Leu41Pro mutation (case1)	mild form; hepatomegaly, growth retardation, no other symptoms	skewed	[41]
c.879G>A	p.Gln293Gln (p.Tyr285Glufs*47)	synonymous mutation and new splicing site (leading to a 28 bp deletion and premature termination	mild form; IDS activity: 1–8.71 nmol/4 h/mg in peripheral leucocytes	skewed > 95:5	[42]
c.1327C>T	p.Arg443Ter	nonsense - premature termination	mild form; short stature, coarse facial features, short neck, mild mental retardation, no other typical symptoms; IDS activity: 1.4 nmol/4 hr/mg protein in fibroblasts and 20.0 nmol/4 hr/mg protein in leukocytes	not determined (*AR* locus noninformative)	[43]
c.1327C>T	p.Arg443Ter	*de novo* mutation; premature termination	mild kyphosis, coarse facial features, contractures of the hands, knee, and ankle joints, limited and painful mobility, recurrent respiratory infections, macrocephaly, coarse facial features, wide eyebrows, flattened nose bridge and anteverted nostrils, short neck, respiratory distress, hepatosplenomegaly, umbilical hernia, hirsurtism, difficulties with active movements of the extremities, severe bilateral hypoacusia, under age performance at school; IDS activity: 9.9–18.29 mmol/h/l dried blood spots	skewed 92:8	[44]
c.1403G>A	p.Arg468Gln	substitution/missense	severe form; coarse face, hearing loss, hepatosplenomegaly, limited joint mobility, retarded development, dysostosis multiplex; urinary GAG: 86 mg uronic acid/g creatinine; excessive amounts of dermatan sulfate and heparan sulfate; IDS activity: not detected in cultured fibroblasts and lymphocytes	skewed	[45]

c.1403G>A	p.Arg468Gln	substitution/missense	severe form; snoring, hearing impairment, delayed speech, coarse facial features, gingival hyperplasia, mild hepatosplenomegaly, sternal protrusion, claw hands, lumbal hyperlordosis, large joint contractures, mild mental retardation, mild paleocerebellar symptoms, mild mitral regurgitation, dysostosis multiplex; urinary GAG: 60.5–65.7 g/mol creatinine; IDS activity: 0.46 nmol/4 h/mg in leukocytes and 19 nmol/4 h/ml in serum	skewed 96:4 to 99:1	[46]
c.1403G>T	p.Arg468Leu	substitution/missense	slightly delayed early motor development, delayed speech development, coarse face, stiff joints, hepatomegaly, skeletal involvement, hyperactivity; urinary GAG: 183 mg uronic acid/g creatinine; excessive amounts of dermatan sulfate and heparan sulfate; IDS activity: not detected in cultured fibroblasts and lymphocytes	skewed	[47]
c.1568A>G	p.Tyr523Cys	substitution/missense	delayed mental development, hyperactivity, joint stiffness, mild scoliosis, hepatomegaly, macrocephaly, coarse facial features, prominent forehead; urinary GAG: 195 mg GAG/gram of creatinine; IDS activity: 35 pmoles/ml/h in serum and 18 pmoles/ mg of protein/18 h in cultured fibroblasts	skewed 97:3	[48]
c.1436_1440del AGCCG (maternal origin) and 46,X,del(X)(q 22.1) (paternal origin)	-	deletion resulting in frame shift and premature termination<br><br>partial deletion of the long arm of paternal X (*IDS* allele disrupted)	Turner syndrome; Hunter syndrome, mild form; growth retardation, lower limbs pain, stiff joints, rough facial features, sunken nose, full lips, eye hypertelorism, macroglossia, short neck, low position of the auricles, stiffness of large and small joints, equino-varus deformity of the feet, kyphosis of thoracic spine, normal intelligence; urinary GAG: increased; IDS activity: 0.001 μM/l/h in dry blood stains	not determined; comorbidity of Turner syndrome (46,X,del(X)(q 22.1) of paternal origin and Hunter syndrome due to deletion in the *IDS* allele of maternal origin	[49]
[c.706_719del + c.705_720insG]	-	frameshift = premature termination	delayed psychomotor and speech development, coarse facial features, macroglossia, hepatosplenomegaly,	skewed	[50]

(c.706_719delins G)			arthrogryposis of fingers and toes, gibbus, short stature, progressive hearing impairment, recurrent ear infections, behavioural problems, insomnia, carpal tunnel syndrome, moderate airway obstruction; urinary GAG: 219.7 mg/creatinine; IDS activity: 1.9 nmol/hour/mg protein in fibroblasts and no activity in leukocytes		
c.1006+1541_1180+1405delins CCACACAGGC ATGAGCCATG (original description: 17419del3254/ 17418ins20)	not determined	*de novo* 3254 bp deletion extending from intron 7 (nt 17419, L35485) to intron 8 (nt 20692, L35485) with an insertion of 20 nucleotides (5'-CCACACAGGC ATGAGCCATG) at position 17418	severe form; coarse face, hearing loss, hepatosplenomegaly, severe thoracolumbar kyphosis, limited joint mobility, mental retardation, mitroaortic valvular dysplasia, but no corneal clouding	skewed	[41]
c.1–103_184del		deletion that includes exons 1–4 of the *IDS* gene (minimal deletion range c.1–103_184del)	severe form: umbilical hernia, slight developmental delay, chronic nasal discharge, coarse facial features, enlarged tongue, stiffness of joints, camptodactyly, hypertrichosis, hoarse voice, hepatosplenomegaly, impaired hearing; urinary GAG: 987 mg/g of creatinine; IDS activity: 2 nmoles/ml/ 4hr in serum and 0.16nmoles/mg of protein/4 hr in leukocytes	skewed<br><br>Her mother and sister had almost totally skewed XCI; however, they were not carriers for the deletion present in the patient.	[51]
46,XX,del(X)(q25) (original description: [del(X)(q25)]) plus unknown *IDS* mutation (in	not determined	deletion not involving the *IDS* allel; unmasking the mutant *IDS* allele inherited from the carrier mother on the normal X chromosome	abdominal distension, hepatomegaly, coarse facies, hirsutism, contraction deformities; urinary GAG: 105 mg/mmol creatinine; marked increases of dermatan and heparan sulphates; IDS activity: less than 2% of control in leucocytes, serum and cultured fibroblasts (0.001–0.07 nmol/h per mg protein)	skewed<br>the X chromosome with deletion is preferentially inactive	[52]

the second allele)					
46,XX,del(X)(q27.3q28) (original description: deletion of the Xq27.3-q28 including partial deletion of the *IDS* gene)	partial IDS deletion not including the putative active site sequence of the enzyme; truncated mRNA from the mutant allele was detected	*de novo* deletion of the paternal X chromosome extending 3–5 cM to the centromeric side of the *IDS* gene	developmental delay, macrocephaly, dysmorphic facies, hypotonia, hepatosplenomegaly, generalized dysostosis multiplex, mucopolysacchariduria	skewed inactivation of the normal maternal *IDS* allel	[53–55]
intragenic inversion	-	intragenic inversion due to a recombination between *IDS* and *ΨIDS* inherited from mother	severe form; organomegaly, mental retardation, behavioural problems, seizures recurrent upper respiratory tract infections, speech delay, hyperactivity, aggressiveness, altered sleep–wake rhythm, coarse facial features, joint stiffness, dysostosis multiplex, minimal mitral valve insufficiency; urinary GAG/creatinine ratio 170,25 x 10^{-3}; IDS activity: 0.24 nmol/4 h/mg in cultured skin fibroblasts	skewed	[56]
46XX,t(X;5) (original description: 46XX,t(X:5))	not determined	*de novo* balanced reciprocal translocation disrupting the *IDS* gene	severe form; urinary GAG: 65 mg/mmol creatinine; IDS activity not detected in fibroblasts	not determined, suspected	[57]
46XX,t(X;9)<br><br>neither genomic *IDS* mutations nor abnormal *IDS* transcripts were detected	not determined	*de novo* balanced reciprocal X;9 translocation<br>possible role of the chromosomal rearrangement in reducing the *IDS* translation efficiency	short stature, angle class III dental malocclusion, faint gingival hypertrophy, brachydactyly, bending of fingers, limited pronation, reduced elbow flexion, and lumbar hyperlordosis, dysostosis multiplex: anisospondyly, converging radius and ulnar distal extremities, small and irregular carpal bones, pointed proximal ends of the second and fifth metacarpal; no intellectual disability and good school performance; urinary GAG: 202.5 mg/g; IDS activity: 3.57 nmol/4 hr/mg protein	skewed 100:0	[58]

not determined; normal karyotype 46,XX	not determined	no data	female 1: coarse facies, speckled blue irises, no corneal clouding, deafness, psychomotor retardation, moderate spasticity, liver enlargement, splenomegaly, died at 7.5 years of age; urinary GAG: elevated excretion; IDS activity: 0.1–0.3 units/g wet tissue in tissue homogenates and 0.02 units/g protein in fibroblast culture female 2: upper respiratory infections, delayed motor development, hepatosplenomegaly, coarse facial features with broad nasal bridge, deafness, tense thick skin; urinary GAG: elevated excretion; IDS activity of 0.4-1.8 units/g wet tissue in homogenates of different tissues	not determined	[59]
not determined; normal karyotype 46,XX	not determined	no data	level of GAGs in the patient's urine (11 mg GAG/mmol creatinine); unusually high amounts of dermatan sulphate; enzyme activity 3.6–6.4 pmol/mg of protein/hr in plasma, not detectable in fibroblasts	skewed	[60]
			**Adrenoleukodystrophy (*ABCD1*)**		
no data	p.Arg74Trp	substitution/missense	EDSS 5.5, gait disorder, spasticity, weakness, pathological reflexes	not determined	[61]
no data	p.Thr105Pro	substitution/missense	DSS: 2.0; EDSS: 2.0; AI: 2	73:27	[62]
no data	p. AsnN148Ser	substitution/missense	disability status scale (DSS): 3.0; the expanded disability status scale (EDSS): 4.0; the ambulation index (AI): 2	100:0	[62]
no data	p.TyrY212Ter	nonsense – premature termination	DSS: 3.0; EDSS: 3.5; AI: 2	93:7	[62]
no data	p.Leu220Pro	substitution/missense	EDSS 4.0; urinary incontinence; faecal incontinence; gait disorder; sensory disturbance; spasticity; weakness; pathological reflexes	not determined	[61]
c.829-832insAAT	-	-	DSS: 3.0; EDSS: 3.5; AI: 1	82:18	[62]
no data	p.Arg401Gln	substitution/missense	DSS: 2.0; EDSS: 2.5; AI: 1	71:29	[62]
no data	p.Gly512Ser	substitution/missense	DSS: 1.0; EDSS: 3.5; AI: 2	92:08	[62]
no data	p.Pro543Leu	substitution/missense	DSS: 3.0; EDSS: 3.0; AI: 3	74:26	[62]

no data	p.Pro543Leu	substitution/missense	EDSS 3.5; gait disorder; sensory complaints; sensory disturbance; weakness; pathological reflexes	not determined	[61]
no data	p.Leu654Pro	substitution/missense	EDSS 6.0; urinary incontinence; faecal incontinence; gait disorder; sensory disturbance; spasticity; weakness; pathological reflexes	not determined	[61]
no data	p.Arg660Trp	substitution/missense	EDSS 3.5; urinary incontinencel; gait disorder; weakness; pathological reflexes	not determined	[61]
no data	p.Arg660Trp	substitution/missense	EDSS 6.0; urinary incontinence; gait disorder; sensory disturbance; weakness; pathological reflexes	not determined	[61]
-	exon8-10del	deletion of exons 8-10	EDSS 3.5; urinary incontinence; faecal incontinence; gait disorder; sensory complaints; sensory disturbance; pathological reflexes	not determined	[61]
**Glucose-6-phosphate dehydrogenase deficiency (*G6PD*)**					
c.95A>G		Gaozhou variant	biochemically G6PD-deficient; age 95 years	74:26	[63]
c.1376G>T		(Canton variant)	presented with hemolysis after cotrimoxizole therapy at the age of 86	97:3	[64]
c.1376G>T			presented with hemolysis after cotrimoxizole therapy at the age of 61	92:3	[64]
c.1376G>T			biochemically G6PD-deficient; age 87 years	79:21	[63]
c.1376G>T			biochemically G6PD-deficient; age 99 years	71:29	[63]

[a] as reported by authors, although in disagreement with the description of a change at protein level.

References

1. Blanco Sánchez, T.; Cañedo Villarroya, E.; Martínez Zazo, A.; Pérez González, B.; Pedrón Giner, C. Hepatic involvement in a female carrier heterozygous for a mutation in the PHKA2 gene. *An. Pediatría (English Ed.* **2016**, *85*, 267–268, doi:10.1016/j.anpede.2016.02.001.

2. Cho, S.Y.; Lam, C. wan; Tong, S.F.; Siu, W.K. X-linked glycogen storage disease IXa manifested in a female carrier due to skewed X chromosome inactivation. *Clin. Chim. Acta* **2013**, *426*, 75–78, doi:10.1016/j.cca.2013.08.026.

3. Willemsen, M.; Rodenburg, R.J.T.; Teszas, A.; van den Heuvel, L.; Kosztolanyi, G.; Morava, E. Females with PDHA1 gene mutations: A diagnostic challenge. *Mitochondrion* **2006**, *6*, 155–159, doi:10.1016/j.mito.2006.03.001.

4. Fujii, T.; Coster, R.N. Van; Old, S.E.; Medori, R.; Winter, S.; Gubits, R.M.; Matthews, P.M.; Brown, R.M.; Brown, G.K.; Dahl, S.H.-H.M.; et al. Pyruvate

Dehydrogenase Deficiency: Molecular Basis for Intrafamilial Heterogeneity. *Ann Neurol* **1994**, *36*, 83–89.

5. Matthews, P.M.; Brown, R.M.; Otero, L.J.; Marchington, D.R.; Legris, M.; Howes, R.; Meadows, L.S.; Shevell, M.; Scriver, C.R.; Brown, G.K. Pyruvate dehydrogenase deficiency. Clinical presentation and molecular genetic characterization of five new patients. *Brain* **1994**, *117*, 435–443, doi:10.1093/brain/117.3.435.

6. Lissens, W.; Vreken, P.; Barth, P.; Wijburg, F.; Ruitenbeek, W.; Wanders, R.; Seneca, S.; Liebaers, I.; De Meirleir, L. Cerebral palsy and pyruvate dehydrogenase deficiency : identification of two new mutations in the E1α gene. *Eur J Pediatr* **1999**, *158*, 853–857.

7. Matthews, P.M.; Brown, R.M.; Otero, L.; Marchington, D.; Leonard, J. V.; Brown, G.K. Neurodevelopmental abnormalities and lactic acidosis in a girl with a 20-bp deletion in the x-linked pyruvate dehydrogenase E1α subunit gene. *Neurology* **1993**, *43*, 2025–2030, doi:10.1212/wnl.43.10.2025.

8. Dahl, H.H.M.; Hansen, L.L.; Brown, R.M.; Danks, D.M.; Rogers, J.G.; Brown, G.K. X-linked pyruvate dehydrogenase E1α subunit deficiency in heterozygous females: Variable manifestation of the same mutation. *J. Inherit. Metab. Dis.* **1992**, *15*, 835–847, doi:10.1007/BF01800219.

9. Horga, A.; Woodward, C.E.; Mills, A.; Pareés, I.; Hargreaves, I.P.; Brown, R.M.; Bugiardini, E.; Brooks, T.; Manole, A.; Remzova, E.; et al. Differential phenotypic expression of a novel PDHA1 mutation in a female monozygotic twin pair. *Hum. Genet.* **2019**, *138*, 1313–1322, doi:10.1007/s00439-019-02075-9.

10. Laróvere, L.E.; Ruiz, S.M.S.; Arranz, J.A.; Kremer, R.D. de Mutation Spectrum and Genotype-Phenotype Correlation in a Cohort of Argentine Patients with Ornithine Transcarbamylase Deficiency: A Single-Center Experience. *J. Inborn Errors Metab. Screen.* 2018, *6*, 1–5.

11. Peng, M.-Z.; Li, X.-Z.; Mei, H.-F.; Sheng, H.-Y.; Yin, X.; Jiang, M.-Y.; Cai, Y.-N.; Su, L.; Lin, Y.-T.; Shao, Y.-X.; et al. Clinical and biochemical characteristics of patients with ornithine transcarbamylase deficiency. *Clin. Biochem.* **2020**, *84*, 63–72, doi:10.1016/j.clinbiochem.2020.06.011.

12. Clarkston, K.; Lee, J.; Donoghue, S.; Peters, H.; Eiroa, H.; Shah, A.A.; Loomes, K.; Wen, J.; Oliver, M.; Hardikar, W.; et al. Acute liver dysfunction with delayed peak of serum aminotransferase levels as a presentation of ornithine transcarbamylase deficiency in females. *Am. J. Med. Genet. A* 2020.

13. Takanashi, J.; Kurihara, A.; Tomita, M.; Kanazawa, M.; Yamamoto, S.; Morita, F.; Ikehira, H.; Tanada, S.; Kohno, Y. Distinctly abnormal brain metabolism in late-onset ornithine transcarbamylase deficiency. *Neurology* **2002**, *59*, 210–214, doi:10.1212/wnl.59.2.210.

14. Nguyen, H.-H.; Khanh Nguyen, N.; Dung Vu, C.; Thu Huong Nguyen, T.; Nguyen, N.-L. Late-Onset Ornithine Transcarbamylase Deficiency and Variable Phenotypes in Vietnamese Females With OTC Mutations. *Front. Pediatr.* 2020, *8*, 321.

15. Gyato, K.; Wray, J.; Huang, Z.J.; Yudkoff, M.; Batshaw, M.L. Metabolic and neuropsychological phenotype in women heterozygous for ornithine transcarbamylase deficiency. *Ann. Neurol.* **2004**, *55*, 80–86, doi:10.1002/ana.10794.

16. Ali, E.Z.; Zakaria, Y.; Mohd Radzi, M.A.; Ngu, L.H.; Jusoh, S.A. Mutation Study of Malaysian Patients with Ornithine Transcarbamylase Deficiency:

Clinical, Molecular, and Bioinformatics Analyses of Two Novel Missense Mutations of the OTC Gene. *Biomed Res. Int.* **2018**, *2018*, 1–15, doi:10.1155/2018/4320831.

17. Caldovic, L.; Abdikarim, I.; Narain, S.; Tuchman, M.; Morizono, H. Genotype-Phenotype Correlations in Ornithine Transcarbamylase Deficiency: A Mutation Update. *J. Genet. Genomics* **2015**, *42*, 181–194, doi:10.1016/j.jgg.2015.04.003.

18. Komaki, S.; Matsuura, T.; Oyanagi, K.; Hoshide, R.; Kiwaki, K.; Endo, F.; Shimadzu, M.; Matsuda, I. Familial lethal inheritance of a mutated paternal gene in females causing X-linked ornithine transcarbamylase (OTC) deficiency. *Am. J. Med. Genet.* **1997**, *69*, 177–181, doi:10.1002/(sici)1096-8628(19970317)69:2<177::aid-ajmg12>3.0.co;2-i.

19. Musalkova, D.; Sticova, E.; Reboun, M.; Sokolova, J.; Krijt, J.; Honzikova, J.; Gurka, J.; Neroldova, M.; Honzik, T.; Zeman, J.; et al. Variable X-chromosome inactivation and enlargement of pericentral glutamine synthetase zones in the liver of heterozygous females with OTC deficiency. *Virchows Arch.* **2018**, *472*, 1029–1039, doi:10.1007/s00428-018-2345-x.

20. Hackett, A.; Gillard, J.; Wilcken, B. n of 1 trial for an ornithine transcarbamylase deficiency carrier. *Mol. Genet. Metab.* **2008**, *94*, 157–161, doi:10.1016/j.ymgme.2008.02.001.

21. Valik, D.; Sedova, Z.; Starha, J.; Zeman, J.; Hruba, E.; Dvorakova, L. Acute hyperammonaemic encephalopathy in a female newborn caused by a novel, de novo mutation in the ornithine transcarbamylase gene. *Acta Paediatr.* **2004**, *93*, 710–711.

22. Ueta, A.; Sumi, S.; Ito, T.; Ban, K.; Hamajima, N.; Togari, H.; Wada, Y.; Kidouchi, K.; Fujimoto, S. Intra-day variations in urinary pyrimidines in ornithine carbamoyltransferase deficiency and healthy individuals. *Clin. Chim. Acta.* 2001, *308*, 187–189.

23. Tuchman, M.; Holzknecht, R.A.; Gueron, A.B.; Berry, S.A.; Tsai, M.Y. Six new mutations in the ornithine transcarbamylase gene detected by single-strand conformational polymorphism. *Pediatr. Res.* **1992**, *32*, 600–604, doi:10.1203/00006450-199211000-00024.

24. Ducamp, S.; Kannengiesser, C.; Touati, M.; Garçon, L.; Guerci-Bresler, A.; Guichard, J.F.; Vermylen, C.; Dochir, J.; Poirel, H.A.; Fouyssac, F.; et al. Sideroblastic anemia: Molecular analysis of the ALAS2 gene in a series of 29 probands and functional studies of 10 missense mutations. *Hum. Mutat.* **2011**, *32*, 590–597, doi:10.1002/humu.21455.

25. Katsurada, T.; Kawabata, H.; Kawabata, D.; Kawahara, M.; Nakabo, Y.; Takaori-Kondo, A.; Yoshida, Y. A Japanese family with X-linked sideroblastic anemia affecting females and manifesting as macrocytic anemia. *Int. J. Hematol.* **2016**, *103*, 713–717, doi:10.1007/s12185-016-1949-7.

26. Qiu, Y.; Cai, H.; Cui, L.; Liu, Y. xian; Wang, Y. ning; Li, J.; Cao, X. xin Identification of a novel heterozygous ALAS2 mutation in a young Chinese female with X-linked sideroblastic anemia. *Ann. Hematol.* 2020, *99*, 371–373.

27. Cazzola, M.; May, A.; Bergamaschi, G.; Cerani, P.; Rosti, V.; Bishop, D.F. *Familial-skewed X-chromosome inactivation as a predisposing factor for late-onset X-linked sideroblastic anemia in carrier females*; 2000;

28. Gonçalves, P.; Pereira, J.C.; Ribeiro, M.L. Gene symbol: ALAS2. Disease: sideroblastic anaemia. *Hum. Genet.* **2004**, *115*, 532.

29. Donker, A.; Raymakers, R.; Nieuwenhuis, H.; Coenen, M.; Janssen, M.; Mackenzie, M.; Brons, P.; Swinkels, D. X-linked sideroblastic anaemia due to $ALAS_2$ mutations in the Netherlands: a disease in disguise. *undefined* **2014**.

30. Aivado, M.; Gattermann, N.; Rong, A.; Giagounidis, A.A.N.; Prall, W.C.; Czibere, A.; Hildebrandt, B.; Haas, R.; Bottomley, S.S. X-linked sideroblastic anemia associated with a novel ALAS2 mutation and unfortunate skewed X-chromosome inactivation patterns. *Blood Cells, Mol. Dis.* **2006**, *37*, 40–45, doi:10.1016/j.bcmd.2006.04.003.

31. Lee, P.L.; Barton, J.C.; Rao, S. V.; Acton, R.T.; Adler, B.K.; Beutler, E. Three kinships with ALAS2 P520L (c. 1559 C → T) mutation, two in association with severe iron overload, and one with sideroblastic anemia and severe iron overload. *Blood Cells, Mol. Dis.* **2006**, *36*, 292–297, doi:10.1016/j.bcmd.2005.12.004.

32. Balwani, M.; Naik, H.; Anderson, K.E.; Bissell, D.M.; Bloomer, J.; Bonkovsky, H.L.; Phillips, J.D.; Overbey, J.R.; Wang, B.; Singal, A.K.; et al. Clinical, biochemical, and genetic characterization of north American patients with erythropoietic protoporphyria and x-linked protoporphyria. *JAMA Dermatology* **2017**, *153*, 789–796, doi:10.1001/jamadermatol.2017.1557.

33. Brancaleoni, V.; Balwani, M.; Granata, F.; Graziadei, G.; Missineo, P.; Fiorentino, V.; Fustinoni, S.; Cappellini, M.D.; Naik, H.; Desnick, R.J.; et al. X-chromosomal inactivation directly influences the phenotypic manifestation of X-linked protoporphyria. *Clin. Genet.* **2016**, *89*, 20–26, doi:10.1111/cge.12562.

34. Møller, L.; Lenartowicz, M.; Zabot, M.T.; Josiane, A.; Burglen, L.; Bennett, C.; Riconda, D.; Fisher, R.; Janssens, S.; Mohammed, S.; et al. Clinical expression of Menkes disease in females with normal karyotype. *Orphanet J. Rare Dis.* **2012**, *7*, doi:10.1186/1750-1172-7-6.

35. Xu, J.; Wang, L.; Liu, X.; Dai, Q. A novel LAMP2 p.G93R mutation associated with mild Danon disease presenting with familial hypertrophic cardiomyopathy. *Mol. Genet. genomic Med.* **2019**, *7*, e00941, doi:10.1002/mgg3.941.

36. Fanin, M.; Nascimbeni, A.C.; Fulizio, L.; Spinazzi, M.; Melacini, P.; Angelini, C. Generalized lysosome-associated membrane protein-2 defect explains multisystem clinical involvement and allows leukocyte diagnostic screening in Danon disease. *Am. J. Pathol.* **2006**, *168*, 1309–1320, doi:10.2353/ajpath.2006.050646.

37. Bottillo, I.; Giordano, C.; Cerbelli, B.; D'Angelantonio, D.; Lipari, M.; Polidori, T.; Majore, S.; Bertini, E.; D'Amico, A.; Giannarelli, D.; et al. A novel LAMP2 mutation associated with severe cardiac hypertrophy and microvascular remodeling in a female with Danon disease: a case report and literature review. *Cardiovasc. Pathol. Off. J. Soc. Cardiovasc. Pathol.* **2016**, *25*, 423–431, doi:10.1016/j.carpath.2016.07.005.

38. Hedberg Oldfors, C.; Mathe, G.; Thomson, K.; Tulinius, M.; Karason, K.; Ostman-Smith, I.; Oldfors, A. Early onset cardiomyopathy in females with Danon disease. *Neuromuscul. Disord.* **2015**, *25*, 493–501, doi:10.1016/j.nmd.2015.03.005.

39. Chen, X.-L.; Zhao, Y.; Ke, H.-P.; Liu, W.-T.; Du, Z.-F.; Zhang, X.-N. Detection of somatic and germline mosaicism for the LAMP2 gene mutation c.808dupG in a Chinese family with Danon disease. *Gene* **2012**, *507*, 174–176, doi:10.1016/j.gene.2012.06.064.

40. Majer, F.; Vlaskova, H.; Krol, L.; Kalina, T.; Kubanek, M.; Stolnaya, L.; Dvorakova, L.; Elleder, M.; Sikora, J. Danon disease: a focus on processing of the novel LAMP2 mutation and comments on the beneficial use of peripheral white blood cells in the diagnosis of LAMP2 deficiency. *Gene* **2012**, *498*, 183–195, doi:10.1016/j.gene.2012.02.004.

41. Cudry, S. MPS II in females: molecular basis of two different cases. *J. Med. Genet.* **2000**, *37*, 29e – 29, doi:10.1136/jmg.37.10.e29.

42. Zhang, H.; Li, J.; Zhang, X.; Wang, Y.; Qiu, W.; Ye, J.; Han, L.; Gao, X.; Gu, X. Analysis of the IDS Gene in 38 Patients with Hunter Syndrome: The c.879G>A (p.Gln293Gln) Synonymous Variation in a Female Create Exonic Splicing. *PLoS One* **2011**, *6*, e22951, doi:10.1371/journal.pone.0022951.

43. Sohn, Y.B.; Kim, S.J.; Park, S.W.; Park, H.-D.; Ki, C.-S.; Kim, C.H.; Huh, S.W.; Yeau, S.; Paik, K.-H.; Jin, D.-K. A mother and daughter with the p.R443X mutation of mucopolysaccharidosis type II: Genotype and phenotype analysis. *Am. J. Med. Genet. A* **2010**, *152A*, 3129–3132, doi:10.1002/ajmg.a.33589.

44. Piña-Aguilar, R.E.; Zaragoza-Arévalo, G.R.; Rau, I.; Gal, A.; Alcántara-Ortigoza, M.A.; López-Martínez, M.S.; Santillán-Hernández, Y. Mucopolysaccharidosis type II in a female carrying a heterozygous stop mutation of the iduronate-2-sulfatase gene and showing a skewed X chromosome inactivation. *Eur. J. Med. Genet.* **2013**, *56*, 159–162, doi:10.1016/j.ejmg.2012.11.006.

45. Sukegawa, K.; Song, X.-Q.; Masuno, M.; Fukao, T.; Shimozawa, N.; Fukuda, S.; Isogai, K.; Nishio, H.; Matsuo, M.; Tomatsu, S.; et al. Hunter disease in a girl caused by R468Q mutation in the iduronate-2-sulfatase gene and skewed inactivation of the X chromosome carrying the normal allele. *Hum. Mutat.* **1997**, *10*, 361–367, doi:10.1002/(SICI)1098-1004(1997)10:5<361::AID-HUMU5>3.0.CO;2-I.

46. Řeboun, M.; Rybová, J.; Dobrovolný, R.; Včelák, J.; Veselková, T.; Štorkánová, G.; Mušálková, D.; Hřebíček, M.; Ledvinová, J.; Magner, M.; et al. X-Chromosome Inactivation Analysis in Different Cell Types and Induced Pluripotent Stem Cells Elucidates the Disease Mechanism in a Rare Case of Mucopolysaccharidosis Type II in a Female. *Folia Biol. (Praha).* **2016**, *62*, 82–9.

47. Sukegawa, K.; Matsuzaki, T.; Fukuda, S.; Masuno, M.; Fukao, T.; Kokuryu, M.; Iwata, S.; Tomatsu, S.; Orii, T.; Kondo, N. Brother/sister siblings affected with Hunter disease: evidence for skewed X chromosome inactivation. *Clin. Genet.* **1998**, *53*, 96–101, doi:10.1111/j.1399-0004.1998.tb02654.x.

48. Kloska, A.; Jakóbkiewicz-Banecka, J.; Tylki-Szymańska, A.; Czartoryska, B.; Węgrzyn, G. Female Hunter syndrome caused by a single mutation and familial XCI skewing: Implications for other X-linked disorders. *Clin. Genet.* **2011**, *80*, 459–465, doi:10.1111/j.1399-0004.2010.01574.x.

49. Semyachkina, A.N.; Voskoboeva, E.Y.; Zakharova, E.Y.; Nikolaeva, E.A.; Kanivets, I. V; Kolotii, A.D.; Baydakova, G. V; Kharabadze, M.N.; Kuramagomedova, R.G.; Melnikova, N. V Case report: a rare case of Hunter syndrome (type II mucopolysaccharidosis) in a girl. *BMC Med. Genet.* **2019**, *20*, 66, doi:10.1186/s12881-019-0807-x.

50. Tuschl, K.; Gal, A.; Paschke, E.; Kircher, S.; Bodamer, O.A. Mucopolysaccharidosis type II in females: Case report and review of literature. *Pediatr. Neurol.* **2005**, *32*, 270–272, doi:10.1016/j.pediatrneurol.2004.10.009.

51. Jurecka, A.; Krumina, Z.; Żuber, Z.; Różdżyńska-Świątkowska, A.; Kłoska, A.; Czartoryska, B.; Tylki-Szymańska, A. Mucopolysaccharidosis type II in females and response to enzyme replacement therapy. *Am. J. Med. Genet. Part A* **2012**, *158A*, 450–454, doi:10.1002/ajmg.a.34415.

52. Broadhead, D.M.; Kirk, J.M.; Burt, A.J.; Gupta, V.; Ellis, P.M.; Besley, G.T.N. Full expression of Hunter's disease in a female with an X-chromosome deletion leading to non-random inactivation. *Clin. Genet.* **1986**, *30*, 392–398, doi:10.1111/j.1399-0004.1986.tb01896.x.

53. Clarke, J.T.; Greer, W.L.; Strasberg, P.M.; Pearce, R.D.; Skomorowski, M.A.; Ray, P.N. Hunter disease (mucopolysaccharidosis type II) associated with unbalanced inactivation of the X chromosomes in a karyotypically normal girl. *Am. J. Hum. Genet.* **1991**, *49*, 289–97.

54. Clarke, J.T.R.; Willard, H.F.; Teshima, I.; Chang, P.L.; Skomorowski, M.A. Hunter disease (mucopolysaccharidosis type II) in a karyotypically normal girl. *Clin. Genet.* **1990**, *37*, 355–362, doi:10.1111/j.1399-0004.1990.tb03519.x.

55. Clarke, J.T.; Wilson, P.J.; Morris, C.P.; Hopwood, J.J.; Richards, R.I.; Sutherland, G.R.; Ray, P.N. Characterization of a deletion at Xq27-q28 associated with unbalanced inactivation of the nonmutant X chromosome. *Am. J. Hum. Genet.* **1992**, *51*, 316–322.

56. Manara, R.; Rampazzo, A.; Cananzi, M.; Salviati, L.; Mardari, R.; Drigo, P.; Tomanin, R.; Gasparotto, N.; Priante, E.; Scarpa, M. Hunter syndrome in an 11-year old girl on enzyme replacement therapy with idursulfase: brain magnetic resonance imaging features and evolution. *J. Inherit. Metab. Dis.* **2010**, *33*, 67–72, doi:10.1007/s10545-009-9023-8.

57. Mossman, J.; Blunt, S.; Stephens, R.; Jones, E.E.; Pembrey, M. Hunter's disease in a girl: association with X:5 chromosomal translocation disrupting the Hunter gene. *Arch. Dis. Child.* **1983**, *58*, 911–915, doi:10.1136/adc.58.11.911.

58. Lonardo, F.; Di Natale, P.; Lualdi, S.; Acquaviva, F.; Cuoco, C.; Scarano, F.; Maioli, M.; Pavone, L.M.; Di Gregorio, G.; Filocamo, M.; et al. Mucopolysaccharidosis type II in a female patient with a reciprocal X;9 translocation and skewed X chromosome inactivation. *Am. J. Med. Genet. Part A* **2014**, *164*, 2627–2632, doi:10.1002/ajmg.a.36667.

59. Neufeld, E.F.; Liebaers, I.; Epstein, C.J.; Yatziv, S.; Milunsky, A.; Migeon, B.R. The Hunter syndrome in females: is there an autosomal recessive form of iduronate sulfatase deficiency? *Am. J. Hum. Genet.* **1977**, *29*, 455–461.

60. Winchester, B.; Young, E.; Geddes, S.; Genet, S.; Hurst, J.; Middelton-Price, H.; Williams, N.; Webb, M.; Habel, A.; Malcolm, S. Female twin with hunter disease due to nonrandom inactivation of the X-chromosome: A consequence of twinning. *Am. J. Med. Genet.* **1992**, *44*, 834–838, doi:10.1002/ajmg.1320440625.

61. Engelen, M.; Kemp, S.; De Visser, M.; Van Geel, B.M.; Wanders, R.J.A.; Aubourg, P.; Poll-The, B.T. X-linked adrenoleukodystrophy (X-ALD): Clinical presentation and guidelines for diagnosis, follow-up and management. *Orphanet J. Rare Dis.* **2012**, *7*, 1–15, doi:10.1186/1750-1172-7-51.

62. Maier, E.M.; Kammerer, S.; Muntau, A.C.; Wichers, M.; Braun, A.; Roscher, A.A. Symptoms in carriers of adrenoleukodystrophy relate to skewed X inactivation. *Ann. Neurol.* **2002**, *52*, 683–688, doi:10.1002/ana.10376.

63. Au, W.Y.; Lam, V.; Pang, A.; Lee, W.M.; Chan, J.L.C.; Song, Y.Q.; Ma, E.S.; Kwong, Y.L. Glucose-6-phosphate dehydrogenase deficiency in female octogenarians, nanogenarians, and centenarians. *Journals Gerontol. - Ser. A Biol. Sci. Med. Sci.* **2006**, *61*, 1086–1089, doi:10.1093/gerona/61.10.1086.

64. Au, W.-Y.; Ma, E.S.K.; Lam, V.M.S.; Chan, J.L.C.; Pang, A.; Kwong, Y.-L. Glucose 6-phosphate dehydrogenase (G6PD) deficiency in elderly Chinese women heterozygous for G6PD variants. *Am. J. Med. Genet.* **2004**, *129A*, 208–211, doi:10.1002/ajmg.a.30213.
