# Supplementary material for: Dosage Compensation in Females with X-Linked Metabolic Disorders
Source: Int J Mol Sci. 2021 Apr 26;22(9):4514. doi: 10.3390/ijms22094514 (PMC8123450; doi:10.3390/ijms22094514)
Supplement: Supplementary file 1 [file ijms-22-04514-s001.zip › Supplementary File_Table S3.pdf]

Table S3. Mutations and severity scores of heterozygous females with Fabry disease.

Mutation		Age (years)	MSSI score[a]					XCI status[b]	Ref.
DNA level	Protein level		Total	G	N	C/V	R		
IVS2+1G>A	-	31	17	5	12	0	0	92:08	[1]
IVS5-2delCA	-	45	37	10	14	1	12	61:39	[1]
		31	29	10	15	0	4	60:40	
c.34del24	-	18	21	5	9	1	6	80:20	[1]
		17	21	7	10	0	4	69:31	
c.59_73del	p.Ala20_Trp24del	24	10	-	-	-	-	R	[2]
		53	31	-	-	-	-	R	
c.71G>A	p.Trp24*	47	40	14	16	10	0	51:49	[3]
		45	43	11	15	9	8	59:41	
		36	27	6	9	8	4	50:50	
		7	3	2	1	0	0	56:44	
c.118C>T	p.Pro40Ser	56	34	-	-	-	-	R	[2]
no data	p.His46Arg	20	16	2	8	2	4	67:33	[1]
no data	p.Cys52Ser	50	20	6	6	8	0	53:47	[1]
c.154T>C	p.Cys52Arg	49	17	-	-	-	-	R	[2]
		29	17	-	-	-	-	R	
		37	29	-	-	-	-	M	
		35	15	-	-	-	-	M	
		66	15	-	-	-	-	W	
		27	8	-	-	-	-	R	
no data	p.Cys63Tyr	28	21	8	13	0	0	51:49	[1]
c.194ins14	-	18	14	3	3	8	0	M	[4]
		22	17	6	3	8	0	M	
c.194+1G>A	unknown	56	17	-	-	-	-	R	[2]
	p.Arg112His	20	1	1	0	0	0	57:43	[1]
		20	25	6	11	0	8	53:47	
c.334C>T	p.Arg112Cys	28	15	-	-	-	-	M	[2]
		68	38	-	-	-	-	M	
no data	p.Leu129Pro	36	29	4	11	14	0	60:40	[1]
no data	p.Leu131Pro	62	32	9	7	8	8	51:49	[1]
c.427G>A	p.Ala143Thr	24	11	-	-	-	-	W	[2]
c.463G>C	p.Asp155His	27	18	2	6	10	0	M	[4]
c.467C>A	p.Ala156Asp	33	4	-	-	-	-	R	[2]
c.547+398insGGTA	unknown	66	38	-	-	-	-	R	[2]
c.548G>A	p.Glu183Asp	67	28	-	-	-	-	R	[2]
		62	25	-	-	-	-	R	
c.593T>C	p.Ile198Thr	37	8	-	-	-	-	R	[2]
		25	10	-	-	-	-	R	
c.606T>G	p.Cys202Trp	64	34	-	-	-	-	M	[2]
c.606_607del	p.Cys202Ter	21	18	-	-	-	-	R	[2]
c.644A>G	p.Asn215Ser	41	9	-	-	-	-	R	[2]
		43	12	-	-	-	-	R	
no data	p.Arg220Ter	14	4	2	2	0	0	84:16	[1]
		67	38	7	13	10	8	83:17	

		54	20	5	7	8	0	70:30	
c.679C>T	p.Arg227Ter	26	2	-	-	-	-	R	[2]
		51	29	-	-	-	-	M	
no data	p.Trp236Cys	68	44	11	9	16	8	76:24	[1]
		37	18	7	7	0	4	76:26	
c.713G>A	p.Ser238Asn	22	3	-	-	-	-	W	[2]
c.717_718delAA	del2(aa)	12	11	1	0	10	0	M	[4]
c.718delAA	-	33	17	5	7	1	4	56:44	[1]
c.744delT	-	3	1	1	0	0	0	84:16	[1]
c.770C>T	p.Ala257Val	34	1	-	-	-	-	R	[2]
		51	7	-	-	-	-	R	
		65	37	-	-	-	-	M	
		53	7	-	-	-	-	W	
		30	1	-	-	-	-	R	
c.778G>A	p.Gly260Arg	63	40	-	-	-	-	R	[2]
c.782dupG	p.Trp262Lysfs*3	63	22	-	-	-	-	NA	[2]
c.797A>T	p.Asp266Val	45	22	-	-	-	-	R	[2]
		54	3	-	-	-	-	W	
		52	20	-	-	-	-	R	
c.801ins36	-	42	40	10	11	11	8	M	[4]
c.803_806delTAGT	p.Lys268Ter	53	47	13	10	20	4	57:43	[3]
		22	29	13	10	2	4	63:37	
		27	21	4	13	0	4	65:35	
		13	23	8	11	0	4	54:46	
c.884T>G	p.Phe295Cys	20	9	-	-	-	-	R	[2]
		56	25	-	-	-	-	R	
c.901C>T	p.Arg301Ter	65	61	-	-	-	-	M	[2]
no data	p.Arg301Ter	17	8	1	5	2	0	76:24	[1]
c.902C<G	p.Arg301Gln	41	26	-	-	-	-	M	[2]
c.912delC	-	63	29	10	6	5	8	77:23	[1]
c.916C>T	p.Gln306Ter	33	15	-	-	-	-	R	[2]
c.950T>C	p.Ile317Thr	9	1	1	0	0	0	M	[4]
no data	p.Asn320Ile	34	22	6	12	0	4	63:37	[1]
no data	p.Gln321Ter	23	11	5	6	0	0	84:16	[1]
c.983G>A	p.Gly328Glu	52	6	-	-	-	-	W	[2]
		27	16	-	-	-	-	R	
c.988C>T	p.Gln330Ter	44	39	9	11	11	8	M	[4]
		49	38	6	3	11	18	M	
	p.Trp340Ter	71	41	9	14	14	4	75:25	[1]
		47	26	3	7	8	8	53:47	
c.1025G>A	p.Arg342Gln	25	7	2	5	0	0	M	[4],
		57	54	13	18	15	8	M	[2]
		59	16	2	3	3	8	W	
		50	13	-	-	-	-	R	
		53	18	-	-	-	-	R	
c.1033_1034del	p.Ser345Argfs*29	68	37	-	-	-	-	R	[2]
c.1045T>C	p.Trp349Arg	67	38	-	-	-	-	R	[2]
no data	p.Ala350Pro	62	36	9	5	14	8	67:33	[1]

		38	26	3	7	12	4	63:37	
c.1055_1056delCT	p.Ala352Aspfs*22	17	21	4	10	3	4	54:46	[3]
		1	6	1	5	0	0	75:25	
c.1057_1058delAT	p.Met353Aspfs*21	51	28	7	10	11	0	62:38	[3]
c.1065C>A	p.Asn355Lys	54	18	-	-	-	-	R	[2]
c.1072G>A	p.Glu358Lys	20	3	-	-	-	-	R	[2]
c.1081_1100del	p.Glu361Argfs*7	66	16	-	-	-	-	R	[2]
c.1086_1098del	p.Arg363Serfs*24	51	25	-	-	-	-	R	[2]
c.1117G>A	p.Gly373Ser	41	15	-	-	-	-	R	[2]
		49	27	-	-	-	-	R	
		48	11	-	-	-	-	R	
c.1145_1149del	p.Cys382Tyrfs*15	47	19	-	-	-	-	M	[2]
c.1187delT	-	8	2	1	1	0	0	60:40	[1]
c.1232G>A	p.Gly411Asp	34	18	-	-	-	-	R	[2]
c.1235_1236delCT	p.Thr412Serfs*38	45	18	11	7	0	0	NA	[3]

[a] The total MSSI score consists of: general (G), neurological (N), cardiovascular (C/V), and renal (R) sub-scores with the maximum values of 18, 20, 20, and 18 respectively; based on MSSI scores, clinical phenotypes are assessed as mild (< 20), moderate (20–40) or severe (> 40) [5].

[b] R, random X chromosome inactivation; M, skewed X-chromosome inactivation with predominant expression of the mutant *GLA* allele; W, skewed X chromosome inactivation with predominant expression of the wild-type *GLA* allele; NA, not available.

References

1. Maier, E.; Osterrieder, S.; Whybra, C.; Ries, M.; Gal, A.; Beck, M.; Roscher, A.; Muntau, A. Disease manifestations and X inactivation in heterozygous females with Fabry disease. *Acta Paediatr.* **2006**, *95*, 30–38, doi:10.1080/08035320600618809.
2. Echevarria, L.; Benistan, K.; Toussaint, A.; Dubourg, O.; Hagege, A.A.; Eladari, D.; Jabbour, F.; Beldjord, C.; De Mazancourt, P.; Germain, D.P. X-chromosome inactivation in female patients with Fabry disease. *Clin. Genet.* **2016**, *89*, 44–54, doi:10.1111/cge.12613.
3. Juchniewicz, P.; Kloska, A.; Tylki-Szymańska, A.; Jakóbkiewicz-Banecka, J.; Węgrzyn, G.; Moskot, M.; Gabig-Cimińska, M.; Piotrowska, E. Female Fabry disease patients and X-chromosome inactivation. *Gene* **2018**, *641*, 259–264, doi:10.1016/j.gene.2017.10.064.
4. Dobrovolny, R.; Dvorakova, L.; Ledvinova, J.; Magage, S.; Bultas, J.; Lubanda, J.C.; Elleder, M.; Karetova, D.; Pavlikova, M.; Hrebicek, M. Relationship between X-inactivation and clinical involvement in Fabry heterozygotes. Eleven novel mutations in the alpha-galactosidase A gene in the Czech and Slovak population. *J. Mol. Med.* **2005**, *83*, 647–654, doi:10.1007/s00109-005-0656-2.
5. Whybra, C.; Kampmann, C.; Krummenauer, F.; Ries, M.; Mengel, E.; Miebach, E.; Baehner, F.; Kim, K.; Bajbouj, M.; Schwarting, A.; et al. The Mainz Severity Score Index: A new instrument for quantifying the Anderson - Fabry disease phenotype, and the response of patients to enzyme replacement therapy. *Clin. Genet.* **2004**, *65*, 299–307, doi:10.1111/j.1399-0004.2004.00219.x.
